# Janus electrospun nanofiber membranes from bio-based furan polyamides for antibacterial wound care

**DOI:** 10.1016/j.bioactmat.2026.06.022

**Published:** 2026-06-24

**Authors:** Xiang Ding, Naing Tun Thet, Carmelo Herdes, Edward Chaloner, Mikal Negasi, Ioanna Kontou, Maisem Laabei, Ute Jungwirth, Dominic Savage, Muhammad Kamran, Michael Zachariadis, Toby Jenkins, Matthew G. Davidson, Hannah S. Leese

**Affiliations:** a Department of Chemistry, University of Bath, Bath, BA2 7AY, UK; b Department of Chemical Engineering, University of Bath, Bath, BA2 7AY, UK; c Department of Life Sciences, University of Bath, Bath, BA2 7AY, UK; d Newcastle Drug Discovery Group, Translational and Clinical Research Institute, Newcastle University, Newcastle, NE2 4HH, UK; e School of Cellular and Molecular Medicine, University of Bristol, Bristol, BS8 1TD, UK; f Imaging Facility, Core Research Facilities, University of Bath, Bath, BA2 7AY, UK

**Keywords:** Electrospinning, Janus membrane, Wound dressing, Antibacterial, Sustainable

## Abstract

Early-stage bacterial contamination and rapid biofilm growth are critical barriers to effective wound healing, highlighting the need for dressing materials that enable prompt, localised antibacterial intervention while maintaining cytocompatibility and sustainability. Here, we report a sustainable electrospun Janus nanofiber membrane based on two bio-derived semi-aromatic furan polyamides, poly(octamethylene furanamide) (PA8F) and poly(decamethylene furanamide) (PA10F), for antibacterial wound dressing applications. Although PA8F and PA10F differ only by two methylene units and show modest wettability differences as dense films, electrospinning into nanofiber networks amplifies this subtle molecular contrast into a pronounced, robust wettability asymmetry that enables a Janus dressing architecture without chemical surface modification. Tetracycline was physically dispersed within the hydrophilic PA8F, prior to electrospinning, to localise antibiotic delivery at the wound-material interface. The Janus membrane exhibits uniform, bead-free nanofibrous morphology and pronounced interfacial wettability asymmetry. Molecular dynamics simulations reveal distinct polymer-water interaction behaviours that underpin the experimentally observed hydration contrast between PA8F and PA10F. Drug release studies demonstrate rapid antibiotic availability, reaching ∼20 μg mL^−1^ in phosphate-buffered saline within 4 h. The Janus membranes achieve ∼1 log and ∼2 log reductions against *Pseudomonas aeruginosa* and *Staphylococcus aureus* colony biofilms, respectively, and produce ∼0.5 log bacterial reduction in an ex vivo porcine burn wound infection model. This study establishes the first use of sustainable furan-based semi-aromatic polyamides as electrospun wound dressings and demonstrates how electrospinning-induced asymmetry can translate subtle molecular differences into efficient, localised antibacterial delivery for advanced wound care.

## Highlights

• Sustainable Janus nanofiber membranes are fabricated from bio-based furan polyamides.
• Electrospinning amplifies subtle chain-length-dependent wettability differences.
• Asymmetric PA8F/PA10F membranes enable rapid local antibiotic delivery with high cytocompatibility.
• Antibacterial efficacy is validated in biofilm and ex vivo porcine wound models.

## Graphical abstract

**Figure:**
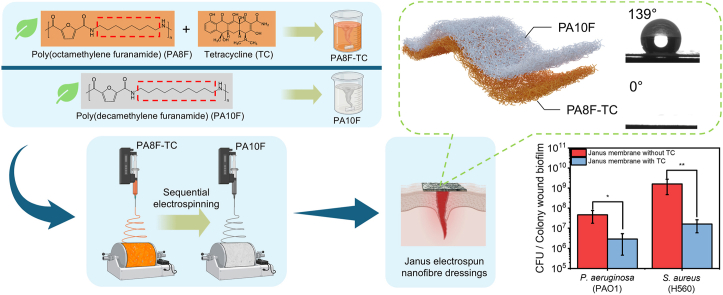

## 1 Introduction

**Scheme 1:**
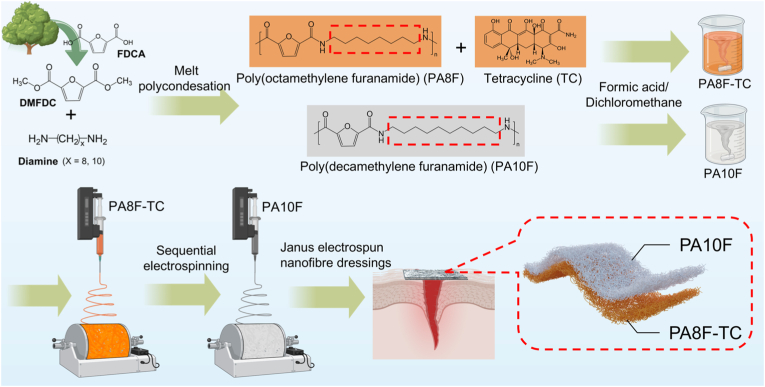
Schematic illustration of the synthesis, processing, and application of the PA8F/PA10F Janus nanofiber membrane. Bio-based semi-aromatic furan polyamides, poly(octamethylene furanamide) (PA8F) and poly(decamethylene furanamide) (PA10F), were synthesized via melt polycondensation using dimethyl 2,5-furandicarboxylate (DMFDC), the dimethyl ester derivative of 2,5-furandicarboxylic acid (FDCA), together with aliphatic diamines. FDCA is a renewable furan-based monomer typically derived from biomass resources. Tetracycline (TC) was dispersed in the PA8F electrospinning solution to generate a drug-containing precursor, while PA10F was prepared as a separate electrospinning solution. Sequential electrospinning was employed to fabricate an asymmetric Janus nanofiber membrane, combining a hydrophilic, drug-releasing PA8F-TC layer with a hydrophobic PA10F barrier layer. The resulting Janus membrane is designed for wound dressing applications, enabling antibacterial delivery while maintaining moisture regulation and protection.

Chronic and infected wounds remain a major clinical challenge, imposing a substantial burden on healthcare systems worldwide [1,2]. In the UK alone, wound management has been estimated to cost the National Health Service on the order of several billion pounds annually, with infection and delayed healing representing major cost drivers [3]. Globally, wound care expenditure has been reported to exceed one hundred billion US dollars per year, underscoring the scale of the problem and the need for improved therapeutic strategies [4] (see Scheme 1).

Clinically, impaired healing is frequently associated with early microbial colonisation followed by biofilm formation and sustained inflammation, which collectively hinder tissue regeneration and increase the risk of local deterioration and systemic complications [5,6]. Biofilms are detected in a substantial fraction of chronic wounds and are widely recognised as a major contributor to treatment recalcitrance once established [7]. Importantly, the transition from initial bacterial contamination to structured biofilm formation can occur within the first few hours after wound exposure, defining a critical early window during which timely antibacterial intervention is most effective. Among wound pathogens, *Staphylococcus aureus* and *Pseudomonas aeruginosa* are repeatedly reported as two of the most commonly isolated organisms in chronic wound infections and are often linked to worsened outcomes [8,9]. These clinical realities motivate wound dressings that not only provide physical protection and moisture management, but also deliver prompt, localised antibacterial activity during the early stages of wound healing, while remaining compatible with host tissue.

Electrospun nanofiber membranes have emerged as promising wound dressing candidates due to their high surface area, interconnected porosity, and extracellular matrix-like architecture [10,11]. These structural attributes support oxygen exchange, exudate handling, and cell attachment, while offering a versatile platform for incorporating and releasing therapeutic agents. Beyond conventional single-function fibrous mats, Janus (asymmetric) membranes have attracted increasing attention in wound care because they can spatially decouple competing requirements within a single dressing [[12], [13], [14]]. In practical applications, a Janus dressing can present a hydrophilic, bioactive interface to the wound bed to promote rapid wetting, intimate contact, and early-stage drug delivery, while maintaining a more hydrophobic outer surface that moderates outward diffusion, limits excessive moisture loss, and provides a protective barrier against external contamination [[15], [16], [17]]. This functional separation is particularly relevant for infected wounds, where rapid local antibiotic availability during the early post-injury period is critical to suppress bacterial colonisation and impede biofilm establishment, while continued barrier function helps stabilise the wound microenvironment.

Alongside Janus architectures, a wide range of bio-based electrospun wound dressings have been developed using polysaccharides, proteins and bio-derived polyesters, typically combined with bioactive agents or stabilization strategies to achieve wet integrity and antibacterial performance. For example, cellulose acetate/gelatin electrospun mats loaded with berberine have been evaluated for diabetic foot ulcer applications in vitro and in vivo, demonstrating antibacterial activity and enhanced wound healing in diabetic animal models [18]. Similarly, bio-based polyester systems such as poly(3-hydroxybutyrate-co-3-hydroxyvalerate) (PHBV) have been processed into electrospun dressings incorporating natural antimicrobials, with reported antibacterial activity and improved diabetic wound repair [19]. Chitosan-based electrospun dressings are also widely explored, but often require formulation engineering or post-crosslinking to improve spinnability and wet-state stability while maintaining antibacterial function [20]. Despite these advances, reported performance metrics and testing stringency vary considerably across the literature. For clarity, representative primary studies and their reported mechanical and antibacterial metrics are summarised in Table S1. To date, many Janus wound dressings have been fabricated from petroleum-derived polymers or synthetic copolymers, such as polyurethanes and polyesters [21,22]. While these materials can deliver effective mechanical and antibacterial performance, there is growing interest in developing Janus dressings from renewable polymer feedstocks to improve sustainability and reduce reliance on fossil-derived materials in disposable healthcare products [23,24]. Here, “bio-based” refers to renewable feedstock sourcing and not the materials’ direct biodegradability.

Semi-aromatic polyamides derived from furan-based monomers constitute a bio-based alternative to conventional petroleum-derived polyamides [[25], [26], [27]]. From a molecular design perspective, these polymers combine robust amide linkages with the polar, rigid furan ring, enabling strong intermolecular interactions and favourable mechanical integrity, while also providing a tunable polarity that can influence interfacial hydration and aqueous interactions [28,29]. Most studies on furan-based polyamides have focused on thermal, mechanical and barrier properties for packaging and engineering applications, whereas their exploration as electrospun biomaterials remains scarce. Importantly, within this polymer family, subtle variations in the aliphatic segment length can alter chain packing, segmental mobility and the balance between polar and hydrophobic domains [30,31]. We hypothesise that electrospinning can act as an “amplifier” that translates subtle, chain-length-dependent hydration differences at the molecular level into large, functionally relevant interfacial contrasts at the porous nanofiber membrane level, providing a materials-by-design route to Janus wound dressings using structurally similar bio-based polymers, avoiding post-fabrication surface modification.

In this work, we report a sustainable electrospun Janus nanofiber membrane composed of two bio-based semi-aromatic furan polyamides with different aliphatic chain lengths: poly(octamethylene furanamide) (PA8F) and poly(decamethylene furanamide) (PA10F). By exploiting chain-length-dependent differences in hydration behaviour that become strongly expressed in a porous nanofibrous architecture, we construct an asymmetric membrane via sequential electrospinning without surface grafting or post-treatment. Tetracycline is incorporated into the hydrophilic PA8F layer to provide rapid, localised antibacterial activity, while the PA10F layer serves as a more hydrophobic counterpart that moderates transport and maintains barrier function. The Janus membrane is systematically evaluated through physicochemical characterization, molecular simulations, drug release measurements, and antibacterial testing spanning in vitro assays, colony biofilms, and an ex vivo porcine burn wound infection model. This study establishes a new application space for sustainable furan-based polyamides in wound care and demonstrates how electrospinning-enabled asymmetry can be harnessed to create functionally integrated dressings for managing early-stage wound infection.

## 2 Materials and methods

### 2.1 Materials

Poly(octamethylene furanamide) (PA8F) and poly(decamethylene furanamide) (PA10F) were synthesized following a previously reported method using dimethyl 2,5-furandicarboxylate (DMFDC) as the furanic diester and octamethylenediamine (OMDA) or decamethylenediamine (DMDA) as the corresponding aliphatic diamines, respectively [25,26]. The resulting polymer was purified and dried prior to use. Formic acid (FA, ≥98%), dichloromethane (DCM, ≥98%), Phosphate Buffered Saline (PBS) pellets and tetracycline (98.0-102.0%) were purchased from Sigma-Aldrich, UK. Human dermal adult fibroblasts were obtained from a commercial supplier and used for in vitro cytocompatibility assessment. Dulbecco's Modified Eagle Medium (DMEM) was purchased from Thermo Fisher Scientific, UK. Cell viability was evaluated using an EZMTT cell viability assay kit purchased from Sigma-Aldrich, UK. All cell culture consumables were tissue culture grade and used as received. Brain Heart Infusion (BHI) agar, Luria Broth (LB), Tryptic Soy Broth (TSB), Biard Parker Agar (BPA), Cetrimide Agar (CA) and polycarbonate membranes (19 mm in diameter with 200 nm in pore diameter) were acquired from Merck, UK. *Pseudomonas aeruginosa* (PAO1 strain) and *Staphylococcus aureus* (H560 strain) were used as wound pathogenic pathogens, donated from Jenkins Biophysical Chemistry Research Group at the University of Bath, UK.

### 2.2 Preparation of electrospun nanofiber membrane

Electrospun Janus nanofiber membranes composed of PA8F and PA10F were fabricated via a sequential electrospinning process. In a typical procedure, the hydrophilic PA8F layer (with or without tetracycline) was electrospun first, followed by deposition of a PA10F layer to form the hydrophobic top surface.

For preparation of the drug-loaded hydrophilic layer, PA8F was first dissolved in formic acid to obtain a homogeneous polymer solution with a concentration of 28% w/v. Separately, tetracycline powder was dispersed in dichloromethane (DCM) at a concentration corresponding to 3 wt% relative to PA8F under magnetic stirring for 30 min to produce a uniform suspension. The tetracycline dispersion was then added dropwise to the PA8F-formic acid solution and stirred for an additional 30 min to ensure homogeneous distribution of tetracycline within the spinning solution. The resulting PA8F-TC suspension was electrospun for 200 min using a single-needle setup onto a rotating drum collector under ambient conditions (22–25 °C, relative humidity 30-40%), with an applied voltage of 25 kV, a flow rate of 6 μL min^−1^, and a tip-to-collector distance of 18 cm, forming the hydrophilic, drug-releasing layer.

Subsequently, PA10F was dissolved in a binary solvent system of formic acid (FA) and dichloromethane (DCM) (1:1 v/v) to obtain a homogeneous polymer solution with a concentration of 30% w/v. The PA10F solution was then electrospun directly onto the pre-formed PA8F-TC layer for 200 min using an applied voltage of 20 kV, a flow rate of 6 μL min^−1^, and a tip-to-collector distance of 12 cm, yielding a bilayer Janus nanofiber membrane with PA10F as the hydrophobic top layer.

For comparison, drug-free Janus membranes were prepared following the same sequential electrospinning protocol, except that PA8F was prepared without tetracycline and electrospun as the bottom layer prior to deposition of the PA10F top layer.

### 2.3 Characterization of electrospun Janus membranes

The morphology of the electrospun nanofiber membranes was examined using a scanning electron microscope (SEM, Hitachi SU3900) operated at an accelerating voltage of 10 kV. Prior to imaging, all samples were sputter-coated with a ∼20 nm thick gold layer using a Quorum Q150TS sputter coater to minimize charging effects.

Fourier-transform infrared (FTIR) spectroscopy was performed using a Bruker INVENIO® spectrometer to analyze the chemical structure of the membranes. Spectra were collected over the range of 4000-500 cm^−1^ with a resolution of 4 cm^−1^, and characteristic absorption bands were used to confirm polymer composition and tetracycline incorporation.

X-ray diffraction (XRD) analysis was conducted in transmission mode using Cu Kα radiation (λ = 1.5406 Å) at an operating voltage of 40 kV and current of 40 mA. Diffraction patterns were recorded over a 2θ range of 2-75° to evaluate the crystalline structure of the electrospun membranes.

Surface wettability was characterized by static water contact angle (WCA) measurements using a contact angle goniometer (Dataphysics OCA 25). A 3 μL droplet of deionized water was deposited onto the membrane surface, and the contact angle was analysed using SCA 20 software. For Janus membranes, measurements were performed independently on each side to assess asymmetric wettability.

Tensile properties of the electrospun membranes were evaluated using a Linkam modular force stage equipped with a tensile testing system (TST350). Rectangular specimens with dimensions of 40 × 10 mm were prepared, and a gauge length of 20 mm was used for all measurements. Tensile tests were conducted at a constant strain rate of 5 mm min^−1^ at room temperature. Cyclic tensile tests were performed to assess durability under repeated sub-failure deformation. Rectangular specimens (initial length: 20 mm) of Janus membranes with and without tetracycline were subjected to strain-controlled triangular-wave loading between 0 and 3% strain (corresponding to a displacement of 0.6 mm). The crosshead speed was 0.3 mm s^−1^, giving a loading frequency of 0.25 Hz, and samples were cycled for 100 cycles at room temperature.

Thermogravimetric analysis (TGA) was conducted on a TA Instruments Discovery TGA 550 thermogravimetric analyzer. Samples were heated from 30 °C to 600 °C at a rate of 10 °C min^−1^ under a nitrogen atmosphere. Differential scanning calorimetry (DSC) was performed using a TA Instruments Discovery DSC 25 under a nitrogen atmosphere. Samples were heated at 10 °C min^−1^ from 10 to 350 °C, cooled to 10 °C at the same rate, and then reheated to 350 °C. The second heating scans were used for thermal analysis to eliminate thermal history effects.

Confocal imaging was performed using a Zeiss LSM 880 confocal laser scanning microscope equipped with an Airyscan detector. An LD C-Apochromat 40×/1.1 W Korr objective lens was used with a scan zoom of 1.8. Samples were excited at 405 nm, and fluorescence emission was collected through a BP 420-480 + BP 495-550 Airyscan emission filters. For each sample, Z-stack images were acquired with an optimal axial step size of 0.23 μm and pixel dimensions defined by the Zeiss ZEN software. Images are presented as maximum-intensity projections of the Z-stacks without post-processing. Fluorescence emission spectra were obtained using the Lambda mode on the same microscope with excitation at 405 nm. Mean fluorescence intensity was recorded in wavelength bins of 8 nm over the range of 420-695 nm.

### 2.4 Molecular dynamics simulations

Coarse-grained molecular dynamics simulations were employed to investigate the molecular origin of the wettability differences between PA8F and PA10F membranes. Simulations were performed using a SAFT-γ Mie coarse-grained framework, which enables predictive modelling of polymer–water interactions without empirical parameter fitting [32].

PA8F and PA10F were modelled using chemically representative interaction sites corresponding to amide, furan, and aliphatic spacer groups, differing only in the length of the aliphatic segment. Polymer slabs were constructed from 1000 repeat units and equilibrated to form dense membranes with well-defined bulk and surface regions. To probe interfacial hydration, a nanoscopic droplet of coarse-grained water was placed adjacent to the polymer surface and the time evolution of water adsorption and penetration was monitored.

Rather than calculating equilibrium contact angles, analysis focused on surface composition, functional group exposure, hydration pathways, and time-resolved interaction energies between water and key polymer functional groups [32,33]. Interaction energies were analysed over the course of the simulations and averaged over the final 2 ns (8–10 ns), where all systems exhibited stable plateaus. All simulations were conducted using GROMACS (v2018.8) [34]. Full simulation details, including coarse-grained mapping schemes, force-field parameters, and additional analyses, are provided in the Supplementary Material.

### 2.5 UV–vis analysis of tetracycline release

The release of tetracycline from the electrospun membranes was quantified by UV-vis spectroscopy using an Agilent Cary 60 spectrophotometer. Membrane samples were cut into square specimens (2.5 × 2.5 cm) and immersed in 10 mL of phosphate buffer solution (PBS). At predetermined time points, the absorbance of the solution was measured using quartz cuvettes, and the TC concentration was determined from a previously established calibration curve.

### 2.6 In vitro cytocompatibility assessment

The cytotoxicity of the electrospun nanofiber membranes was evaluated using an indirect extract method. Briefly, after sterilizing both sides of the membranes under ultraviolet light, membrane samples were incubated in RPMI-1640 (Merck, R8758) supplemented with 10% foetal bovine serum (FBS) at 37 °C for 24 h (1 membrane punch/600 μl). Human dermal adult fibroblasts were seeded in 96-well plates in RPMI with FBS. After 24 h, the cells were exposed to control or conditioned medium for 72 h. Proliferation was measured using the CyQUANT Direct Cell Proliferation Assay (Thermo Fisher Scientific, C35012) according to the manufacturer's instructions. Cell viability was assessed using an EZMTT assay (MilliporeSigma, CBA410). Data were normalised to cells cultured in RPMI plus FBS alone. Cell viability ≥70% was considered non-cytotoxic. Brightfield images were taken on an incucyte at the 72 h timepoint. IL-6 levels were measured using the IL6 Quantikine ELISA Kit (R&D Systems, Cat#D6050B). In brief, after the 72 h exposure to the extraction medium, cells were washed with PBS and a serum free medium was added and collected after 24 h to assess IL6 levels according to the manufacturer's instructions.

### 2.7 Zones of inhibition (ZOI) assay

*Staphylococcus aureus* strain SH1000 was incubated on tryptic soy agar (TSA) at 37 °C for 18 h. Membrane preparations were tested for antimicrobial activity with zones of inhibition (ZOI) determined via agar diffusion adapted from Clinical and Laboratory Standards Institute methods (CLSI M07-A9). In summary, bacterial cultures were inoculated in 2 mL Tryptic soy broth (TSB) and incubated shaking at 180 rpm at 37 °C for 18 h. Overnight cultures were sub-cultured in TSB and grown to exponential phase by shaking at 180 rpm at 37 °C until they reached an absorbance (OD600 nm) of 0.5-0.6. For determination of ZOI, TSA plates were spread with approximately 5 × 10^5^ CFU mL^−1^ from aliquots of 0.5 McFarland standardised inoculum to generate bacterial lawn plates. Membrane preparations were cut to uniform dimensions and placed on the agar plates, whilst controls utilised 50 μL of either PBS or Tetracycline at 1 mg mL^−1^ incubated onto 6 mm diffusion disks. Plates were incubated at 37 °C for 18 h, and the ZOI were measured to ascertain antimicrobial activity.

### 2.8 In vitro colony biofilm model

An in vitro colony biofilm model was employed to evaluate the antibacterial efficacy of the Janus membranes against early-stage biofilm formation. This model mimics wound biofilms by allowing bacteria to grow on a nanoporous polycarbonate membrane while accessing nutrients from an underlying agar substrate [35].

*Pseudomonas aeruginosa* (PAO1) and *Staphylococcus aureus* (H560) were used as representative wound pathogens. Single bacterial colonies were inoculated into Luria Broth (LB) or Tryptic Soy Broth (TSB), respectively, and incubated at 37 °C with shaking at 200 rpm for 18 h. The overnight cultures were diluted in sterile MilliQ water (pH 7.3), and the bacterial concentration was adjusted to approximately 5 × 10^5^ CFU mL^−1^.

Polycarbonate membranes (19 mm diameter, 200 nm pore size) were placed on Brain Heart Infusion (BHI) agar plates and sterilized by exposure to UV light (254 nm) for 10 min. Each membrane was treated with 20 μL of sterile artificial wound fluid and allowed to dry prior to inoculation with 20 μL of the bacterial suspension. The inoculated plates were incubated at 37 °C under 80% relative humidity for 4 h to allow initial biofilm establishment.

Janus membranes, with or without tetracycline loading, were cut to 19 mm diameter, supported on transparent tape, and placed directly onto the developing colony biofilms. The assemblies were incubated for an additional 4 h under the same conditions. Following incubation, the Janus membranes were removed, and each polycarbonate membrane was transferred into an Eppendorf tube containing 0.5 mL sterile MilliQ water. Biofilm cells were detached by vortexing for 2 min followed by sonication in a water bath for 15 min. The resulting suspensions were serially diluted, plated onto BHI agar, and incubated at 37 °C for 18 h for viable cell quantification by colony counting.

### 2.9 Ex vivo porcine burn wound infection model

An ex vivo porcine burn wound infection model was used to evaluate the antibacterial performance of the Janus membranes under physiologically relevant tissue conditions [36]. Fresh organic porcine belly skin was obtained from a local farm and processed aseptically in a laminar flow cabinet. The subcutaneous fat layer was removed to obtain a skin thickness of approximately 5 mm. The tissue was rinsed thoroughly with deionized water and cut into square sections (approximately 2.5 × 2.5 cm^2^). Skin sections were disinfected using UVC irradiation for two cycles (10 min exposure followed by 10 min rest per cycle) and then transferred to sterile Petri dishes containing sterile gauze wetted with 0.15% (w/v) Virkon in MilliQ water.

Partial-thickness scald burns were created using sterilized brass blocks (contact area 2.0 × 2.0 cm^2^) pre-heated to 170 °C. The heated block was applied to the skin for 10 s to generate second-degree burn wounds. After 10 min, each wound was inoculated with 50 μL of bacterial suspension (approximately 2 × 10^6^ CFU mL^−1^) prepared in sterile MilliQ water. The infected tissues were incubated at 37 °C under 80% relative humidity for 6 h to allow initial bacterial colonisation.

Following colonisation, Janus membranes (with or without tetracycline loading) supported on transparent tape were cut to match the skin section size (2.5 × 2.5 cm^2^) and applied directly onto the wound surface. A gentle additional load was applied to ensure intimate and continuous membrane-wound contact during incubation. Samples were further incubated for 18 h at 37 °C and 80% relative humidity, giving a total infection period of 24 h.

After incubation, membranes were removed and excess wound fluid was gently absorbed using sterile absorbent pads. The wound surface was then thoroughly swabbed using sterile wound swabs. Swabs were vortexed in sterile MilliQ water to recover bacteria, followed by serial dilution and plating for viable cell enumeration. *P. aeruginosa* (PAO1) and *S. aureus* (H560) were quantified using Cetrimide Agar and Baird Parker Agar, respectively, after incubation at 37 °C for colony counting.

## 3 Results and discussion

### 3.1 Physicochemical characterization of PA8F/PA10F Janus nanofiber membranes

**Fig. 1:**
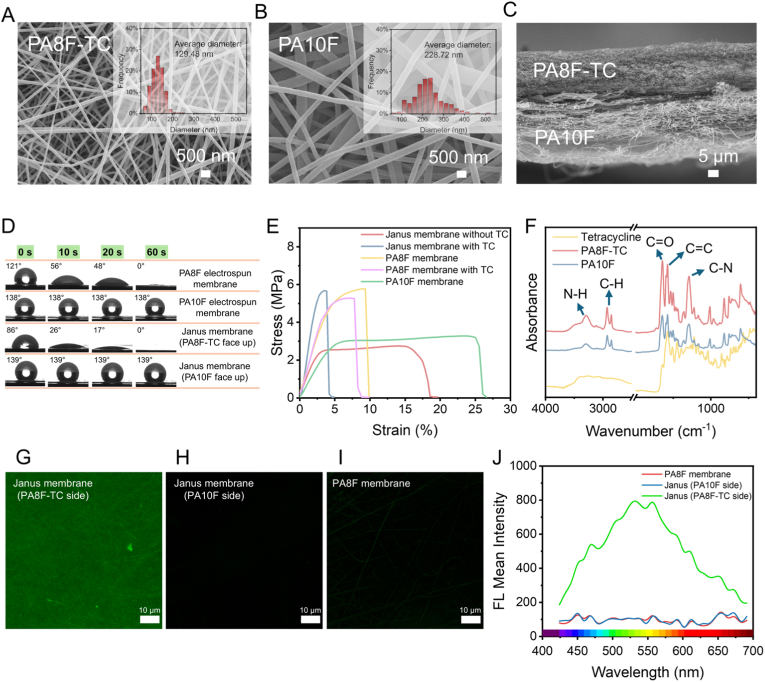
Structural and physicochemical characterization of the PA8F/PA10F Janus nanofiber membranes. SEM images of electrospun membranes (A) PA8F–TC, (B) PA10F and (C) cross-section of the Janus membrane. (D) Dynamic apparent water contact angle (3 μL droplet) and uptake behaviour on electrospun PA8F and PA10F membranes and on each side of the Janus membrane. (E) Tensile stress-strain curves of pristine PA8F, PA8F-TC and PA10F membranes, and Janus membranes with/without TC. (F) FTIR spectra of tetracycline, PA8F-TC, and PA10F membranes. Confocal fluorescence image of the (G) PA8F-TC side, (H) PA10F side of Janus membrane and (I) PA8F membrane. (J) Fluorescence emission spectra extracted from the PA8F membrane, PA10F and PA8F-TC side of the Janus membrane.

The electrospun PA8F, PA8F-TC and PA10F nanofiber membranes exhibit uniform, bead-free morphologies (Fig. 1A and B and Fig. S1). Quantitative analysis of SEM images using ImageJ reveals a systematic difference in fiber scale, with the mean diameter increasing from PA8F (112.61 ± 22.74 nm) to PA8F-TC (129.48 ± 29.29 nm) and PA10F (228.72 ± 72.30 nm). These measurements indicate that tetracycline incorporation slightly increases the fiber diameter of the PA8F layer, while PA10F forms substantially thicker fibers under the selected spinning conditions.

The bilayer Janus architecture is confirmed by the cross-sectional SEM image (Fig. 1C), where two well-defined fibrous layers corresponding to PA8F-TC and PA10F can be clearly identified. Under identical electrospinning duration and flow rate, the two layers exhibit comparable thicknesses, with the PA8F-TC and PA10F layers measuring 21.3 ± 1.3 μm and 23.2 ± 2.2 μm, respectively, resulting in an approximately 50/50 thickness ratio across the membrane. This controlled bilayer structure provides a robust platform for integrating asymmetric interfacial properties into a single membrane.

PA8F and PA10F are structurally closely related semi-aromatic furan polyamides, differing only by two methylene units in the aliphatic diamine segment. This subtle variation in chain length intrinsically affects chain packing, segmental mobility, and the balance between polar amide groups and hydrophobic aliphatic domains. In dense, non-porous hot-pressed films, however, these molecular-level differences are only weakly expressed at the surface. Consistent with this, water contact angle measurements on hot-pressed PA8F and PA10F films reveal only a modest difference, with static contact angles of approximately 78° and 85°, respectively (Supplementary Fig. S2).

The subtle chemical difference becomes markedly amplified when the polymers are processed into electrospun nanofiber membranes. On porous electrospun fiber membranes, the value measured at the moment of droplet deposition represents an apparent contact angle that can be elevated by air entrapment within the porous network. Here, “hydrophilic” is used to describe rapid wetting and water uptake of the porous electrospun membrane, rather than a single instantaneous contact-angle value measured immediately after droplet deposition. The electrospun PA8F membrane shows rapid wetting and imbibition, with the apparent contact angle decreasing from ∼121° to ∼56° within 10 s followed by complete absorption, whereas PA10F maintains a stable apparent contact angle of ∼138° with no noticeable absorption over 60 s (Fig. 1D). These results indicate that while the wettability contrast originates from intrinsic polymer chemistry, electrospinning-induced architecture (high surface area and capillary pathways) amplifies the difference into a pronounced functional asymmetry. Compared with pristine PA8F, tetracycline incorporation further enhances wetting of the PA8F-TC layer, consistent with the polar functional groups present in the antibiotic. In addition to intrinsic polymer architecture, the finer fiber diameter of PA8F/PA8F-TC may further accelerate capillary imbibition and thus amplify the dynamic wetting contrast observed between PA8F and PA10F in the electrospun architecture.

It is worth noting that most reported Janus electrospun wound dressings create wettability asymmetry through either post-fabrication surface modification or by combining highly dissimilar polymers to impose compositional and morphological asymmetry [37,38]. While effective, these strategies can introduce additional processing steps and interfacial complexity, and the long-term stability of the wettability contrast may depend on coating uniformity, surface ageing, or the integrity of blended/phase-separated interfaces under wet conditions. In contrast, the present system exploits molecular-level asymmetry encoded in two structurally similar bio-based furanic polyamides, where a subtle monomer chain-length difference provides an intrinsic and design handle for wettability control. This materials-by-design route reduces reliance on post-treatment and highly dissimilar blends and provides a clearer structure-property rationale for constructing robust Janus functionality.

Mechanical properties were assessed by monotonic tensile testing (Fig. 1E and Table 1). Among the single-component membranes, PA8F shows relatively high strength and stiffness with limited extensibility (UTS 5.78 MPa, EAB 9.34%, and Young's modulus 121.5 MPa). Incorporation of tetracycline causes only modest changes in the PA8F layer (PA8F-TC: UTS 5.29 MPa, EAB 7.79%, and Young's modulus 130.4 MPa), suggesting slight stiffening and reduced ductility. Together with the SEM-derived fiber diameters, this comparison indicates that tetracycline incorporation slightly increased fiber diameter and altered the tensile response of the PA8F-based membrane.

**Table 1:** Fiber diameter and tensile properties of electrospun PA8F, PA10F and Janus membranes.

Sample	Mean diameter (nm)	UTS (MPa)	EAB (%)	Young's Modulus (MPa, 0-2.1% strain)
PA8F	112.61 ± 22.74	5.78	9.34	121.5
PA8F-TC	129.48 ± 29.29	5.29	7.79	130.4
PA10F	228.72 ± 72.30	3.28	25.02	66.2
Janus (no TC)	—	2.75	15.03	89.0
Janus-TC	—	5.68	3.85	227.3

UTS: ultimate tensile strength; EAB: elongation at break. Young's Modulus was obtained from linear fitting of the initial 0-2.1% strain region.

PA10F exhibits lower strength and stiffness but markedly higher elongation (UTS 3.28 MPa, EAB 25.02%, and Young's modulus 66.2 MPa). However, because PA8F and PA10F differ in both fiber morphology and polymer architecture, the observed mechanical differences between these two membranes cannot be attributed to fiber diameter alone.

For the bilayer constructs, the drug-free Janus membrane shows intermediate mechanical behaviour (UTS 2.75 MPa, EAB 15.03%, and Young's modulus 89.0 MPa), whereas tetracycline loading substantially increases stiffness and reduces ductility in Janus-TC (UTS 5.68 MPa, EAB 3.85%, and Young's modulus 227.3 MPa). This pronounced stiffening and loss of extensibility is consistent with restricted fiber-fiber sliding and stress concentration introduced by the dispersed drug phase within the bilayer network. Although the elongation at break of Janus-TC is lower than that of native human skin [39], the membranes retain sufficient strength for handling and fixation.

To place these values in the context of existing wound dressings, polyurethane-based film dressings typically report tensile strengths in the ∼1.7-34 MPa range, and a Tegaderm™ film modulus on the order of ∼9 MPa has been reported in the literature [40,41]. While direct quantitative comparison is not one-to-one due to fundamental differences in structure (porous electrospun nonwovens versus continuous films) and testing conditions, these benchmarks indicate that Janus-TC is relatively stiff and less extensible, which may reduce conformability in highly mobile anatomical sites.

Accordingly, we assessed durability under repeated low-strain deformation using cyclic tensile testing (3% strain, 100 cycles, 0.25 Hz). Both Janus and Janus-TC exhibited stable and repeatable stress responses without evidence of mechanical failure or progressive loss of load-bearing capacity (Fig. S3), supporting robustness under mild dynamic loading and handling. Nevertheless, the low elongation at break of Janus-TC suggests potential limitations in high-strain regions (e.g., joints), where larger deformations and edge stresses could increase the risk of tearing; in such cases, additional fixation or pairing with a more compliant secondary dressing would be required.

All polymer membranes exhibit characteristic absorption bands associated with amide groups and furan rings following FTIR spectroscopy (Fig. 1F), indicating that the polymer backbone remains intact after electrospinning. No distinct new absorption bands attributable to tetracycline can be clearly identified in the PA8F-TC spectrum. This similarity is expected given the relatively low tetracycline loading and the overlap between tetracycline vibrations and the strong amide-related bands of PA8F. Importantly, no significant peak shifts or new bands indicative of chemical bonding are observed, suggesting that tetracycline is physically incorporated within the PA8F nanofibers rather than chemically bound, which is favourable for subsequent drug release.

The XRD patterns of the PA8F and PA10F electrospun membranes, as well as both sides of the Janus membrane, exhibit broad diffraction halos without distinct crystalline peaks, indicating that tetracycline incorporation and Janus assembly do not induce significant changes in the polymer crystalline structure (Supplementary Fig. S4). The Janus membranes with and without tetracycline exhibit nearly identical TGA profiles, indicating that TC incorporation does not significantly affect thermal stability. This is attributed to the low TC content relative to the polymer matrix, which is insufficient to produce a detectable change in the overall thermal decomposition behaviour (Supplementary Fig. S5). DSC thermograms of PA8F, PA10F, and the Janus membranes show comparable thermal behaviour, with a pronounced low-temperature endothermic feature more evident for PA8F, consistent with its higher moisture affinity. The Janus membranes with and without tetracycline exhibit nearly identical second-heating profiles, indicating that the low TC loading does not measurably affect the bulk thermal transitions (Supplementary Fig. S6).

To directly visualise the spatial distribution of tetracycline within the Janus membrane, confocal laser scanning microscopy was performed using high-resolution Airyscan imaging. When excited at 405 nm, a strong and homogeneous fluorescence signal is observed exclusively on the PA8F-TC side of the Janus membrane (Fig. 1G), whereas the opposite PA10F side and pristine PA8F exhibit only background-level fluorescence (Fig. 1H and 1I). This clear contrast confirms that tetracycline remains selectively confined within the hydrophilic PA8F nanofibrous region following sequential electrospinning.

To further substantiate this asymmetric localisation, fluorescence emission spectra were acquired in Lambda mode and quantitatively analysed (Fig. 1J). The PA8F-TC side displays a pronounced emission band spanning approximately 450-600 nm, consistent with the intrinsic fluorescence signature of tetracycline, while both the PA10F side of the Janus membrane and the drug-free control PA8F membranes show negligible emission across the same spectral range. The use of Z-stack acquisition and maximum-intensity projection ensures that the observed fluorescence originates from throughout the nanofibrous layer rather than from superficial adsorption.

These confocal results provide direct evidence that the Janus membrane preserves a well-defined compositional asymmetry at the microscale, with minimal interlayer migration of tetracycline prior to release. This spatial confinement is critical for establishing directional wetting and controlled drug transport and underpins the release behaviour and antibacterial performance.

### 3.2 Insights into chain-length-dependent hydration from molecular dynamics

To elucidate the molecular origin of the experimentally observed wettability contrast between PA8F and PA10F nanofiber membranes, coarse-grained (CG) molecular dynamics simulations based on the SAFT-γ Mie framework were performed. Given the close chemical similarity between PA8F and PA10F, differing only by two methylene units in the aliphatic spacer, the simulations were designed to probe how this subtle structural variation influences surface organisation and hydration behaviour at the molecular level.

**Fig. 2:**
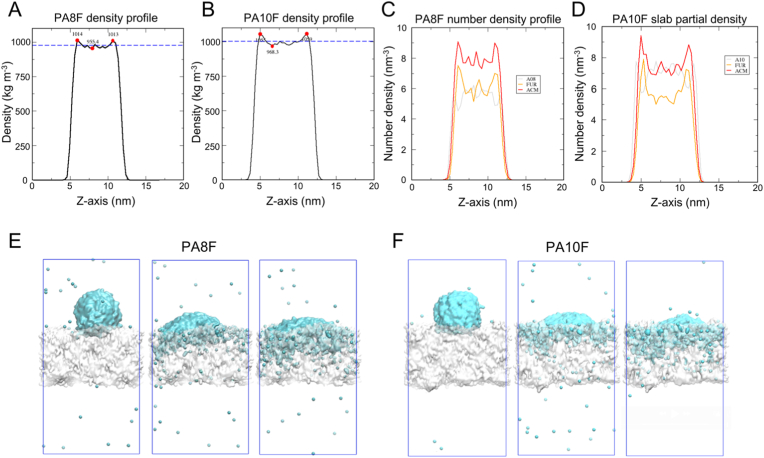
Coarse-grained molecular dynamics simulations revealing chain-length-dependent surface organisation and hydration behaviour of pristine PA8F and PA10F polymer slabs. (A,B) Mass density profiles of PA8F and PA10F slabs along the z-axis, showing homogeneous bulk regions and well-defined polymer-vacuum interfaces. (C,D) Number density profiles of coarse-grained bead types along the z-axis, indicating enrichment of polar acetamide and furan units at the PA8F surface, and increased exposure of aliphatic spacer units at the PA10F surface. (E,F) Representative simulation snapshots illustrating water droplet wetting and infiltration on PA8F and PA10F surfaces at 0 (left), 1 (middle) and 2 (right) ns.

The equilibrium mass density profiles of PA8F and PA10F polymer slabs along the surface normal (z-axis) (Fig. 2A and B) show that both polymers self-assemble into dense, homogeneous slabs with well-defined bulk regions and sharp polymer-vacuum interfaces, confirming that the simulation protocol yields physically stable surfaces. The predicted bulk densities of PA8F (∼977 kg m^−3^) and PA10F (∼1002 kg m^−3^) are comparable, indicating that differences in hydration behaviour cannot be attributed to large variations in bulk packing density.

Despite similar bulk densities, pronounced differences emerge when analysing surface composition. The number density profiles of individual coarse-grained bead types (Fig. 2C and D) reveal distinct interfacial organisation. For PA8F, the surface region is enriched in polar acetamide (AMC) and furan (FUR) beads, while the aliphatic spacer beads (A08) preferentially reside in the interior of the slab. In contrast, PA10F exhibits increased exposure of aliphatic A10 beads at the surface, with a larger fraction of furan units buried within the bulk. This chain-length-dependent segregation indicates that the shorter aliphatic spacer in PA8F allows polar functional groups to more readily populate the interface, whereas the longer spacer in PA10F partially shields these groups and promotes a more hydrophobic surface composition (Fig. S7).

The consequences of this molecular organisation become evident upon water exposure, as illustrated by representative snapshots of nanoscopic water droplet interactions with PA8F and PA10F surfaces (Fig. 2E and F, also see Supplementary Material Fig. S8, Video S1). Upon landing, both systems exhibit similar initial wetting behaviour. However, as the simulations progress, water rapidly wets and penetrates the polymer surface in both cases on sub-nanosecond timescales, precluding the reliable definition of an equilibrium contact angle.

Supplementary video related to this article can be found at https://doi.org/10.1016/j.bioactmat.2026.06.022

The following is the supplementary data related to this article:

Multimedia component 2

Qualitative differences emerge when comparing equivalent simulation times, particularly the configuration at 2 ns. By this point, water has infiltrated the PA8F slab earlier and more extensively, penetrating more than half of the slab thickness, whereas penetration into PA10F remains delayed and spatially constrained, reaching only around one third of the slab. This behaviour is consistent with the higher availability of polar interaction sites at the PA8F interface, while in PA10F the surface is dominated by exposed hydrophobic aliphatic groups. Representative trajectories and movies supporting these observations are provided in the Supplementary material.

**Fig. 3:**
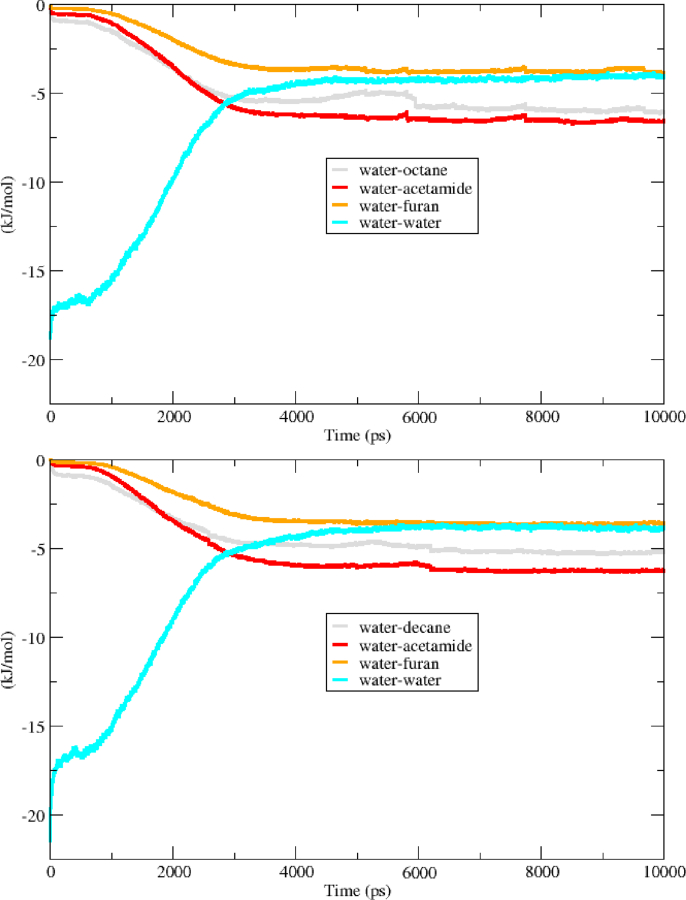
Time evolution of interaction energies between water and representative functional groups for (Top) PA8F and (Bottom) PA10F systems. Interaction energies are shown for water-aliphatic segments (octane/decane), water-acetamide, water-furan, and water-water interactions. All systems exhibit equilibration within the first 4-6 ns, followed by stable plateaus used for averaging (8-10 ns).

To further quantify these differences, the time evolution of interaction energies between water and representative functional groups (aliphatic segments, acetamide groups, and furan units), as well as water–water interactions, was analysed (Fig. 3 and Table 2).

**Table 2:** Averaged interaction energies between water and representative functional groups for PA8F and PA10F systems, obtained from coarse-grained molecular dynamics simulations. Values are reported as mean ± standard deviation, calculated over the final 2 ns (8-10 ns) of the trajectories following equilibration. Interaction energies are shown for water-aliphatic segments (octane/decane), water-acetamide groups, water-furan units, and water-water interactions.

Interaction Type	PA8F (kJ mol^−1^)	PA10F (kJ mol^−1^)
Water–aliphatic	−5.97 ± 0.02	−5.23 ± 0.01
Water–acetamide	−6.55 ± 0.03	−6.24 ± 0.01
Water–furan	−3.73 ± 0.03	−3.59 ± 0.01
Water–water	−4.05 ± 0.02	−3.83 ± 0.02

In both systems, the interaction energies exhibit clear equilibration behaviour, reaching stable plateaus after approximately 4-6 ns. Averaged over the final 2 ns of the simulations (8-10 ns), the water-acetamide interaction is consistently more favourable (more negative) than interactions with aliphatic or furan groups, reflecting the polar nature of the amide functionality, see Table 2.

A comparison between PA8F and PA10F reveals subtle but consistent differences in interaction energetics. In particular, PA8F exhibits slightly more favourable water-acetamide interactions and a more pronounced reduction in water-water interaction energy over time, consistent with enhanced interaction between water and the polymer interface. In contrast, PA10F shows comparatively less favourable water-polymer interactions, consistent with a greater contribution of hydrophobic aliphatic segments at the surface.

These results provide a quantitative energetic perspective that complements the structural analysis, supporting the conclusion that PA8F presents a more hydration-favourable interface, while PA10F exhibits a comparatively more hydrophobic surface character. While absolute wettability metrics such as contact angles are not directly computed within the coarse-grained framework, these relative interaction energies provide a consistent proxy for comparing water affinity across the two polymer systems.

Taken together, the simulations identify a clear molecular mechanism underpinning the experimentally observed wettability contrast. Although PA8F and PA10F possess nearly identical chemical backbones and similar bulk properties, small differences in aliphatic chain length drive pronounced differences in surface functional group exposure. PA8F presents a more polar, hydration-favourable interface, while PA10F forms a comparatively hydrophobic surface. This chain-length-dependent surface reorganisation provides a molecular-scale explanation for the amplified wettability differences observed experimentally in electrospun nanofiber membranes and underpins the asymmetric hydration behaviour exploited in the Janus membrane design.

### 3.3 Time-dependent and directional tetracycline release from Janus nanofiber membranes

**Fig. 4:**
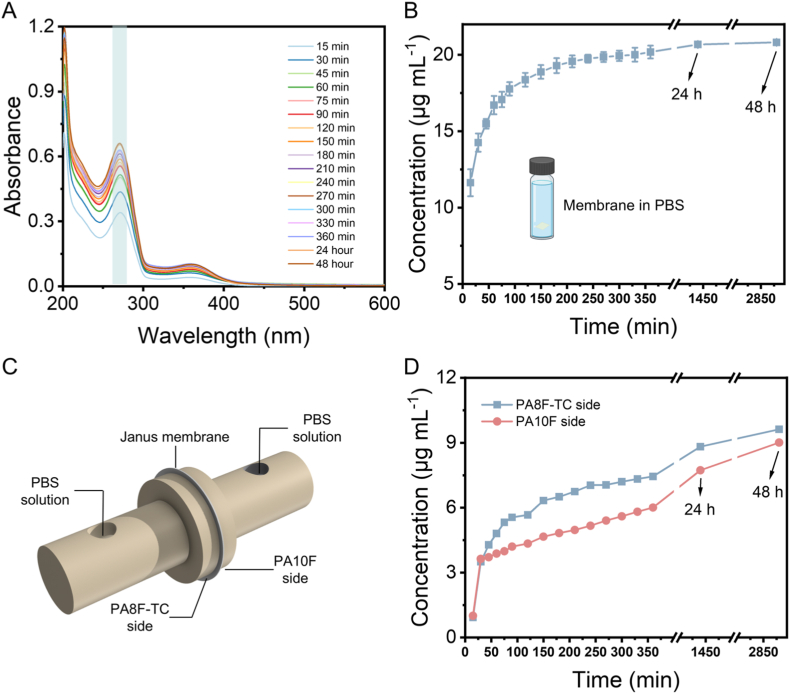
(A) UV–vis absorption spectra of PBS solutions containing the Janus membrane (2.5 × 2.5 cm^2^, ∼6 mg in 10 mL PBS) recorded at different incubation times. (B) Tetracycline concentration in PBS as a function of time. (C) Schematic illustration of a custom-designed 3D-printed dual-chamber device used to evaluate directional tetracycline transport across the Janus membrane. (D) Tetracycline concentration measured in PBS collected from the PA8F–TC side and PA10F side over time.

The release behaviour of tetracycline from the Janus membrane was first evaluated by immersing a membrane sample (2.5 × 2.5 cm^2^, ∼6 mg) in 10 mL of phosphate-buffered saline (PBS) at 37 °C. UV–vis absorption spectra of the release medium were recorded at predetermined time intervals up to 48 h (Fig. 4A). Tetracycline concentration was quantified using a calibration curve obtained from standard solutions (Supplementary Fig. S9), based on the characteristic absorbance peak at 275 nm. The tetracycline concentration in PBS increases rapidly during the early stage of incubation (Fig. 4B), reaching a plateau within approximately 240 min (4 h). The concentration approaches ∼20 μg mL^−1^ and remains relatively stable over extended incubation times up to 48 h. This rapid initial release is attributed to the high hydrophilicity of the PA8F-TC layer combined with the porous nanofibrous architecture, which promotes fast water penetration and diffusion-driven transport of physically incorporated tetracycline. The subsequent plateau indicates that the readily accessible drug fraction is largely exhausted within the first few hours, consistent with a surface- and near-surface-dominated release process rather than long-term sustained diffusion from the polymer bulk.

To further examine the directional transport of tetracycline arising from the asymmetric Janus architecture, a custom-designed dual-chamber device was employed (Fig. 4C). In this configuration, the Janus membrane was fixed between two identical chambers, with the PA8F-TC side and PA10F side simultaneously immersed in equal volumes of PBS (Supplementary Fig. S10). Independent sampling of the PBS from each chamber enabled direct comparison of tetracycline concentration on both sides of the membrane over time.

A clear asymmetry in tetracycline concentration is observed at early time points, with the PA8F-TC side exhibiting significantly higher concentrations than the PA10F side (Fig. 4D and Fig. S11 in Supplementary Material). This behaviour is consistent with preferential release from the hydrophilic, drug-loaded PA8F layer and restricted transport across the more hydrophobic PA10F layer. With increasing incubation time, the concentration difference between the two sides gradually equilibrates, indicating progressive redistribution of tetracycline across the membrane. After 24 h, the concentrations reach 8.8 and 7.7 μg mL^−1^ on the PA8F–TC and PA10F sides, respectively, and further converge to 9.6 and 9.0 μg mL^−1^ after 48 h.

From a wound care perspective, this release behaviour is highly relevant. Rapid antibiotic availability during the first few hours is critical for suppressing early bacterial colonisation and preventing biofilm establishment at the wound interface [42]. In the Janus membrane, the hydrophilic PA8F-TC layer ensures fast and efficient drug release toward the wound, while the opposing PA10F layer acts as a diffusion-limiting barrier that temporarily retards antibiotic transport across the membrane.

This asymmetric structure does not aim to enforce permanent unidirectional release, but rather to introduce an early-stage concentration bias and reduce premature drug loss away from the wound-facing side. Such directional control enables high local antibiotic availability at the wound surface while retaining the drug within the dressing during the critical early phase of healing. These results demonstrate that the Janus architecture provides a rational means to decouple rapid wound-side delivery from outward diffusion control.

### 3.4 In vitro cytocompatibility and antibacterial activity of the Janus membrane

**Fig. 5:**
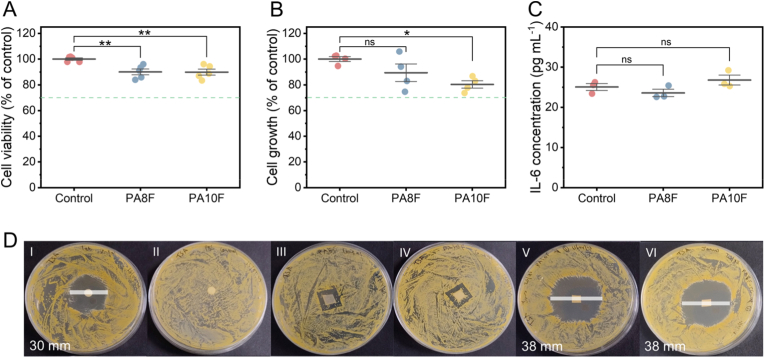
(A) Cell viability of fibroblasts after 72 h exposure to membrane extracts, expressed as % of the control. The dashed line indicates the commonly accepted cytotoxicity threshold (70%). (B) Fibroblast proliferation after 72 h exposure to membrane extracts, assessed by CyQUANT proliferation assay expressed as % of the control. The dashed line indicates the commonly accepted cytotoxicity threshold (70%). (C) IL-6 concentration in fibroblast culture medium after exposure to membrane extracts. (D) Representative zone of inhibition (ZOI) images against *S. aureus*: (I) tetracycline (positive control); (II) PBS (negative control); (III) PA8F membrane without tetracycline; (IV) PA10F membrane without tetracycline; (V) tetracycline-loaded Janus membrane with the PA8F side facing the agar surface; and (VI) tetracycline-loaded Janus membrane with the PA10F side facing the agar surface.

The cytocompatibility results demonstrate that both PA8F and PA10F membranes support primary fibroblast viability over 72 h. Fibroblasts exposed to membrane extracts maintained metabolic activity (Fig. 5A) and their proliferating capacity (Fig. 5B). All measured values remained above the commonly accepted cytotoxicity threshold of 70%. To assess whether the exposure to the membranes could trigger an inflammatory response, IL6 levels in conditioned medium were assessed. No significant differences between control and membrane extract-exposed fibroblasts were detected (Fig. 5C). Furthermore, fibroblasts maintained their morphology throughout the experiments (Fig. S12). Together, these data indicate no overt cytotoxicity under the tested extract conditions and no notable differences between the two semi-aromatic furan polyamides PA8F and PA10F extracts. The antibacterial performance of the membranes against *S. aureus* was assessed using a zone of inhibition assay, with representative results shown in Fig. 5D. As expected, the positive control (tetracycline) produced a clear zone of inhibition, whereas the negative control (PBS) showed no detectable antibacterial effect (Fig. 5D I and II). Pristine PA8F and PA10F membranes without tetracycline did not generate inhibition zones, confirming that the polymer matrices themselves do not possess inherent antibacterial activity (Fig. 5D III and IV). In contrast, tetracycline-loaded Janus membranes produced pronounced inhibition zones, demonstrating effective release of bioactive tetracycline into the surrounding agar. Comparable inhibition zones were observed irrespective of membrane orientation; this behaviour is consistent with the rapid and isotropic diffusion of small-molecule antibiotics in agar-based assays (Fig. 5D V and VI).

### 3.5 Biofilm inhibition and ex vivo wound infection

The antibacterial performance of the Janus membrane was further evaluated using both an in vitro colony biofilm model and an ex vivo porcine wound infection model to assess its efficacy under increasingly complex and physiologically relevant conditions.

**Fig. 6:**
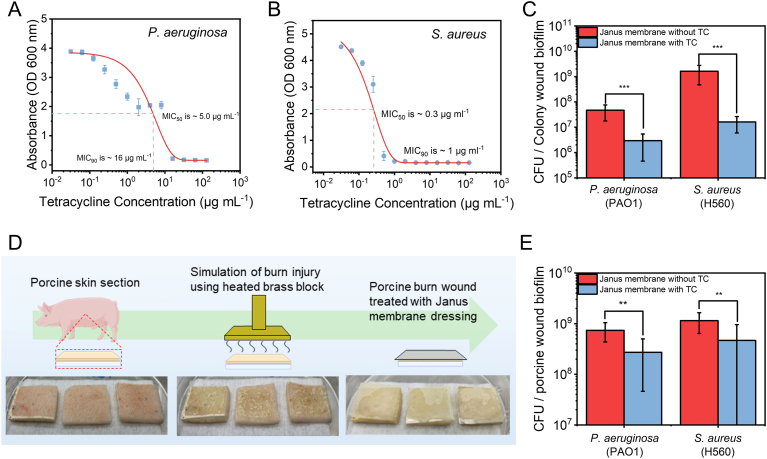
Biofilm inhibition and ex vivo antibacterial performance of the Janus membrane. Determination the MIC_50_ and MIC_90_ for (A) PAO1 and (B) H560 strains using bacterial growth curve in the presence of varying concentrations of tetracycline. (C) Viable cell counts (CFU per biofilm) of PAO1 and H560 after treatment with Janus membranes with and without tetracycline, showing approximately 1 log and 2 log reductions, respectively, *p* = 0.0003(∗∗∗), *p* = 0.0007(∗∗∗). (D) Representative photographs illustrating porcine skin preparation, burn wound induction, and application of the Janus membrane dressing. (E) Viable bacterial counts recovered from infected porcine burn wounds treated with Janus membranes with and without tetracycline, *p* = 0.002(∗∗), *p* = 0.01(∗∗).

The intrinsic susceptibility of the target bacterial strains to tetracycline was first established to contextualise the membrane-based antibacterial results. Growth-curve-based MIC analysis (Supplementary material) indicates concentration-dependent inhibition for both *P. aeruginosa* (PAO1) and *S. aureus* (H560) (Fig. 6A and B). PAO1 exhibits substantially higher tolerance to tetracycline, with MIC_50_ and MIC_90_ values of approximately 5.0 and 16 μg mL^−1^, respectively, whereas H560 shows markedly greater sensitivity, with MIC_50_ and MIC_90_ values of ∼0.3 and ∼1 μg mL^−1^. These values are consistent with literature reports and reflect the well-known intrinsic resistance of Gram-negative *P. aeruginosa* associated with its outer membrane barrier and efflux mechanisms [43].

Building on these susceptibility results, the antibacterial efficacy of the Janus membranes was further examined using an 8 h colony biofilm model to mimic early-stage surface-associated biofilm formation (Supplementary Materials Fig. S13). In this model, bacteria grow on nanoporous polycarbonate membranes supported by nutrient agar, closely resembling wound-associated biofilm development. After an initial 4 h biofilm establishment period, Janus membranes with or without tetracycline were placed in direct contact with the developing biofilms for a further 4 h prior to viable cell quantification.

Janus membranes without tetracycline did not significantly affect biofilm viability for either bacterial strain, indicating that the polymer matrix alone does not disrupt established biofilms (Fig. 6C). In contrast, tetracycline-loaded Janus membranes induced a pronounced reduction in viable biofilm cells for both PAO1 and H560. Quantitatively, approximately 1 log reduction in CFU per biofilm was observed for PAO1, while a more substantial ∼2 log reduction was achieved for H560. The lower biofilm reduction observed for PAO1 is consistent with its higher MIC values relative to H560, demonstrating that the biofilm inhibition performance of the Janus membrane directly reflects the intrinsic tetracycline susceptibility of the target bacteria.

It is noted that the colony biofilm experiments were conducted under aqueous conditions rather than PBS. To account for this, tetracycline release from the Janus membrane into water was independently quantified using an MIC-based assay (Supplementary Fig. S14), revealing that approximately 2.56 μg mL^−1^ of tetracycline is released from 1 cm^2^ of Janus membrane. This release level is comparable to that observed in PBS and lies within the concentration range required to inhibit H560 and partially suppress PAO1, providing consistency between the release studies and the observed biofilm inhibition.

To further validate the antibacterial performance of the Janus membrane under tissue-relevant conditions, an ex vivo porcine burn wound infection model was employed. This model provides a physiologically more realistic environment by incorporating the structural complexity and biochemical components of skin tissue. Representative images illustrating porcine skin preparation, burn wound induction using a heated brass block, and application of the Janus membrane dressing are shown in Fig. 6D.

The antibacterial efficacy of the Janus membranes in the ex vivo model was quantified by enumerating viable bacteria recovered from infected burn wounds after treatment (Fig. 6E). Compared with wounds treated with Janus membranes without tetracycline, tetracycline-loaded Janus membranes resulted in an approximately 0.5 log reduction in viable bacterial counts for both *P. aeruginosa* (PAO1) and *S. aureus* (H560). These results demonstrate that tetracycline released from the Janus membrane remains biologically active and capable of reducing bacterial burden in a complex tissue environment.

Although the extent of bacterial reduction in the ex vivo model was lower than that observed in the in vitro colony biofilm model, this result is expected given the increased complexity of the tissue environment, including heterogeneous surface topology, higher organic load, and restricted antibiotic diffusion. Importantly, the ability of the Janus membrane to reduce bacterial burden under these challenging conditions demonstrates its functional relevance beyond simplified in vitro assays.

We compare our findings with published Janus antibacterial membranes/dressings and the infection models used in Table S3. It suggests that introducing sustainability-driven material choices does not necessarily compromise antibacterial efficacy: multiple Janus platforms achieve clinically relevant antibacterial effects via different mechanisms, and our bio-based furan-polyamide Janus-TC achieves measurable reductions in bacterial burden under stringent colony biofilm and ex vivo tissue conditions. Compared with more engineered Janus systems that incorporate additional functional components, our approach prioritises a simpler bio-based bilayer enabled by intrinsic wettability asymmetry, while maintaining meaningful early-stage infection suppression. This provides a practical balance between sustainability, design simplicity, and antibacterial performance.

Although PA8F and PA10F are derived from bio-based furanic feedstocks, bio-based origin does not necessarily imply biodegradability (as petrochemical origin of a polymer does not preclude biodegradability). Based on the known hydrolytic stability of polyamides, substantial biodegradation under typical environmental, composting, or physiological conditions is not expected, although, to our knowledge, biodegradation/compostability of PA8F/PA10F has not yet been evaluated. More realistic end-of-life options for this class of bio-based polyamides include mechanical and chemical recycling routes. Recent studies on sustainable polyamide platforms report favourable life-cycle impacts together with demonstrated recyclability [27], and life cycle assessment (LCA) and techno-economic analyses (TEA) of furan-derived polymer value chains has also been critically assessed [44]. In clinical practice, used dressings are typically managed via regulated healthcare waste streams.

## 4 Conclusion

In this work, we demonstrate the first use of bio-based semi-aromatic furan polyamides as electrospun wound dressing materials, establishing a previously unexplored biomedical application for this sustainable polymer family. The key conceptual advance is that electrospinning-induced architecture amplifies intrinsic, chain-length-dependent hydration differences between closely related furanic polyamides into a robust wettability asymmetry. This amplification is the enabling mechanism underpinning our Janus wound-dressing design and supports the first demonstration of furanic polyamides as electrospun dressing substrates. Molecular simulations first elucidated the distinct hydration behaviours of PA8F and PA10F, providing a molecular-level rationale for the experimentally observed wettability contrast. Exploiting this effect, a Janus membrane was constructed by sequential electrospinning without chemical modification, integrating a hydrophilic PA8F drug-releasing layer with a more hydrophobic PA10F barrier layer. Incorporation of tetracycline into the hydrophilic PA8F layer did not compromise fiber morphology, while enabling rapid antibiotic availability, reaching ∼20 μg mL^−1^ in PBS within 4 h. The resulting Janus membranes achieved ∼1 log and ∼2 log reductions against *P. aeruginosa* and *S. aureus* colony biofilms, respectively, and produced an approximately 0.5 log reduction in bacterial burden in an ex vivo porcine burn wound model.

Rather than increasing antibiotic loading, the Janus architecture improves delivery efficiency by localising release toward the wound-facing interface, while the PA10F layer moderates transport and limits uncontrolled diffusion. Together, these results establish a new biomedical application for sustainable furan polyamides and demonstrate how electrospinning-induced asymmetry can be harnessed to design efficient antibacterial wound dressings with reduced reliance on systemic antibiotic administration.

## Ethics approval and consent to participate

The procurement and use of ex vivo porcine skin was approved by the Animal Welfare and Ethical Review Body (AWERB) at the University of Bath reference 12774-15028.

## CRediT authorship contribution statement

**Xiang Ding:** Conceptualization, Data curation, Formal analysis, Investigation, Methodology, Writing – original draft. **Naing Tun Thet:** Formal analysis, Investigation, Methodology, Validation. **Carmelo Herdes:** Data curation, Formal analysis, Resources, Software, Validation, Writing – original draft. **Edward Chaloner:** Investigation, Validation. **Mikal Negasi:** Data curation, Investigation, Visualization. **Ioanna Kontou:** Investigation, Validation. **Maisem Laabei:** Formal analysis, Validation. **Ute Jungwirth:** Formal analysis, Validation. **Dominic Savage:** Resources. **Muhammad Kamran:** Resources. **Michael Zachariadis:** Investigation. **Toby Jenkins:** Investigation, Resources. **Matthew G. Davidson:** Funding acquisition, Investigation, Resources. **Hannah S. Leese:** Conceptualization, Funding acquisition, Methodology, Resources, Supervision, Writing – review & editing.

## Declaration of competing interest

The authors declare that they have no known competing financial interests or personal relationships that could have appeared to influence the work reported in this paper.
