# Supplementary material for: Janus electrospun nanofiber membranes from bio-based furan polyamides for antibacterial wound care
Source: Bioact Mater. 2026 Jun 24;65:1043–55. doi: 10.1016/j.bioactmat.2026.06.022 (PMC13316687; doi:10.1016/j.bioactmat.2026.06.022)
Supplement: Multimedia component 1 [file mmc1.docx]

**Supplementary Materials**

**Janus electrospun nanofiber membranes from bio-based furan polyamides for antibacterial wound care**

Xiang Ding^a^, Naing Tun Thet^a^, Carmelo Herdes^b^, Edward Chaloner^c^, Mikal Negasi^d^, Ioanna Kontou^c^, Maisem Laabei^e^, Ute Jungwirth^c,d^, Dominic Savage^a^, Muhammad Kamran^b^, Michael Zachariadis^f^, Toby Jenkins^a^, Matthew G. Davidson^a^, Hannah S. Leese^b*^

^a^Department of Chemistry, University of Bath, Bath, BA2 7AY, UK

^b^Department of Chemical Engineering, University of Bath, Bath, BA2 7AY, UK

^c^Department of Life Sciences, University of Bath, Bath, BA2 7AY, UK

^d^Newcastle Drug Discovery Group, Translational and Clinical Research Institute, Newcastle University, Newcastle, NE2 4HH, UK

^e^School of Cellular and Molecular Medicine, University of Bristol, Bristol, BS8 1TD, UK

^f^Imaging Facility, Core Research Facilities, University of Bath, Bath, BA2 7AY, UK

*corresponding author: h.s.leese@bath.ac.uk

**Table S1.** Summary of electrospun wound dressings based on bio-based materials.

| **Bio-based material system** | **Active component** | **Biological evaluation** | **Mechanical properties** | **Sustainability** | **Ref.** |
| --- | --- | --- | --- | --- | --- |
| Cellulose acetate/gelatin | Berberine + GA vapor crosslink | **Antibacterial:** time-kill CFU vs S. aureus/P. aeruginosa (24 h CFU: 70±3.7 and 44±5.7). **Cytocompatibility:** MTT (L929; reported as biocompatible). **In vivo:** STZ-diabetic rat DFU; collagen density 88.8±6.7%, angiogenesis 19.8±3.8. | 2.83±0.08 MPa (CA/Gel), 2.69±0.05 MPa (CA/Gel/Beri) | Bio-based matrix: Yes; Synthetic carrier polymer: No; Biodegradability: Tested; End-of-life: NR; Process complexity: High | [1] |
| Cellulose acetate | Bioactive glass nanoparticles | **Antibacterial:** ZOI (mm) vs Gram+/− (e.g., E. coli 34±3.2; S. aureus 35±2.3). **Cytocompatibility:** topical skin irritation check in rats (no erythema etc., reported). **In vivo:** diabetic rat; complete closure by day 10; remaining wound areas reported. | - | Bio-based matrix: Yes; Synthetic carrier polymer: No; Biodegradability: NR; End-of-life: NR; Process complexity: Medium | [2] |
| PLA/gelatin | EGF conjugation (bioactive; reported antibacterial effect) | **Antibacterial:** ZOI (mm) vs S. aureus 19±1.2; E. coli 22±1.8. **Cytocompatibility:** MTT (L929; reported; dose-dependent). **In vivo:** wound closure; 99±2.18% reduction at day 18. (PLA/GEL/EGF). | 4.7±0.2 MPa (PLA/GEL/EGF). Young’s modulus reported up to 8.6±0.7 MPa (cross-section). | Bio-based matrix: Yes; Synthetic carrier polymer: No; Biodegradability: NR; End-of-life: NR; Process complexity: Medium | [3] |
| Chitosan/silk fibroin electrospun bilayer | Antimicrobial peptide CM11; GA vapour crosslinking | **Antibacterial:** disk diffusion vs standard + MDR strains (S. aureus, E. coli, P. aeruginosa; inhibition zones reported). **Cytocompatibility:** MTT (Hu02 fibroblasts; non-cytotoxic for ≤32 µg/mL up to 7 d; 64 µg/mL cytotoxic). **In vivo:** NR. | 4.15±0.19 MPa (CS/SF bilayer). | Bio-based matrix: Yes; Synthetic carrier polymer: No; Biodegradability: Tested; End-of-life/circularity: NR; Process complexity: High | [4] |
| Chitosan/HTCC/PEO | HTCC (cationic) ± caffeic acid / berberine; GA vapour crosslinking | **Antibacterial:** survival ratio assay. S. aureus: chitosan-only 72.37% survival; HTCC-containing membranes ~0% survival. E. coli: S6-0X 20.1% survival; caffeic acid system ~0% (12 h); berberine system 19.82% survival. **Cytocompatibility:** NR. **In vivo:** NR. | Crosslinked membranes: 2.7–3.0 MPa; un-crosslinked S1-CU: 3.56 MPa. | Bio-based matrix: Partial; Synthetic carrier polymer: Yes; Biodegradability: NR; End-of-life: NR; Process complexity: High | [5] |
| Chitosan aqueous salt/PVA (e.g., CS-EDTA/PVA) | Intrinsic chitosan antibacterial (salt-dependent) | **Antibacterial:** MIC/MBC vs S. aureus/E. coli (e.g., 30/70 CS-EDTA/PVA MIC=MBC=5.0 mg/mL). **Cytocompatibility:** ISO10993-5 style extract MTT (NHF; non-toxic reported). **In vivo:** rat wound healing; closure within 10 d (all groups), improved early healing noted. | Tensile strength range 8.9–1.5 MPa (depends on CS/PVA ratio); neat PVA 12.8 MPa. | Bio-based matrix: Partial; Synthetic carrier polymer: Yes; Biodegradability: NR; End-of-life: NR; Process complexity: Medium | [6] |
| Zein protein nanofibers | Silver sulfadiazine (0.3–0.6 wt%) | **Antibacterial:** disk diffusion vs E. coli and Bacillus (reported; ZOI values NR in text). **Cytocompatibility:** NR. **In vivo:** NR. | - | Bio-based matrix: Yes; Synthetic carrier polymer: No; Biodegradability: NR; End-of-life: NR; Process complexity: Medium | [7] |
| PHBV (bio-based polyester), radially oriented + berberine | Berberine encapsulation (1% & 5%) | **Antibacterial:** killing rate (5% Beri) >99% for S. aureus, >99% for E. coli, >99% for C. albicans. **Cytocompatibility:** MTT (HDF) + cytokines by ELISA (IL-6/TNF-α reported). **In vivo:** diabetic mouse; wound closure ~100% (5% Beri) at day 18. | (5% Beri) 1.2±0.2 MPa; 56.9±10.0 MPa. | Bio-based matrix: Yes; Synthetic carrier polymer: No; Biodegradability: Claimed; End-of-life: NR; Process complexity: Medium | [8] |
| Furanic polyamides (This work) | Tetracycline-loaded PA8F layer + intrinsic wettability asymmetry | **Antibacterial:** zone of inhibition (*S. aureus*), **colony biofilm model** (*P. aeruginosa* PAO1 ~1 log CFU reduction; *S. aureus* H560 ~2 log CFU reduction), **ex vivo porcine wound model** (~0.5 log CFU reduction vs drug-free Janus). **Cytocompatibility**: fibroblast viability (≥70%), fibroblast growth (CyQUANT); inflammatory response: IL-6 ELISA. | Janus-TC: tensile strength 5.68 MPa; Young’s modulus 227.3 MPa (Drug-free Janus: 2.75 MPa; 89.0 MPa). | Bio-based matrix: Yes; Synthetic carrier polymer: No; Biodegradability: Not claimed; End-of-life: Discussed (recycling/LCA context); Process complexity: Medium | This work |


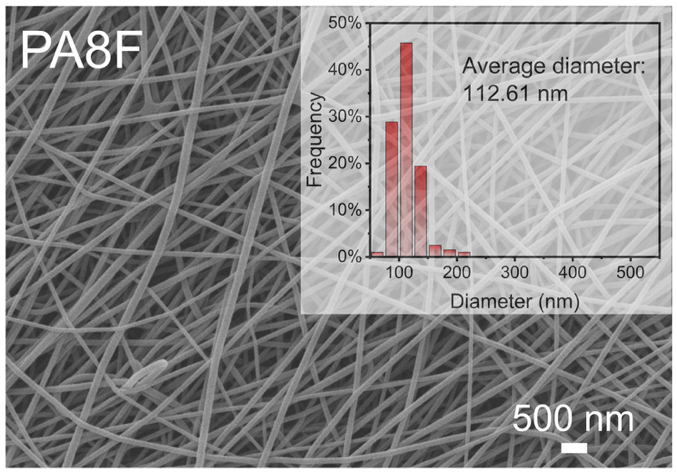


**Fig. S1.** SEM images of PA8F membrane (without tetracycline).


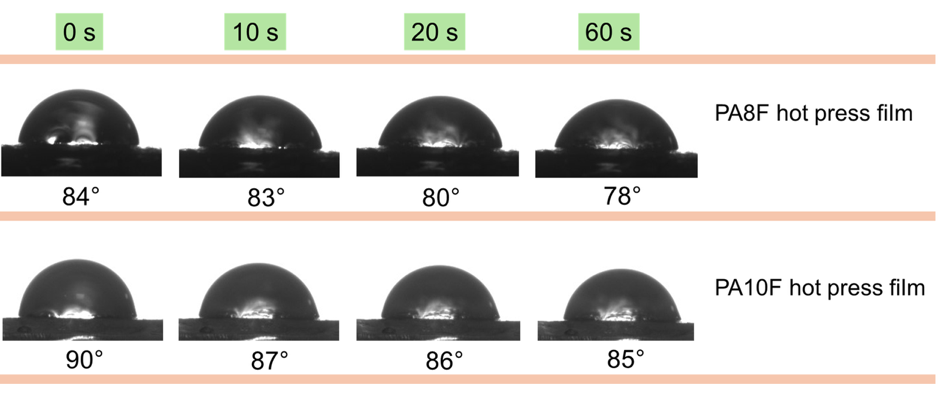


**Fig. S2.** Time-dependent water contact angle images of PA8F and PA10F hot-pressed films.


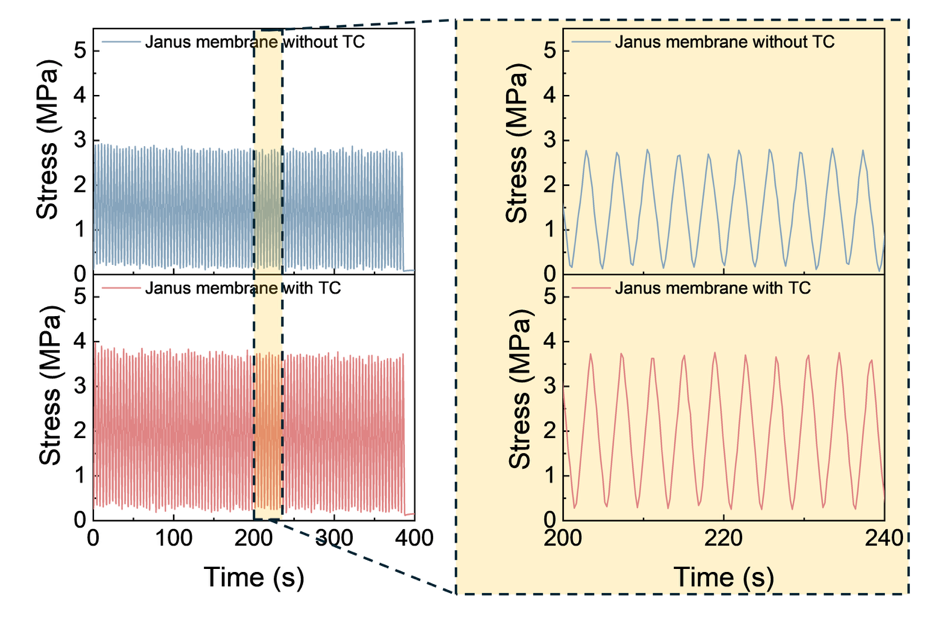


**Fig. S3.** Cyclic tensile response of Janus membranes with and without tetracycline. Samples (20 mm initial length) were cycled between 0 and 3% strain (0.6 mm displacement) at 0.25 Hz for 100 cycles. The right panels show an enlarged time window highlighting the repeatability of the cyclic stress response.

**Fig. S4.** X-ray diffraction (XRD) patterns of PA8F, PA10F electrospun membrane, and the two sides of the PA8F-TC/PA10F Janus membrane.

All samples display broad diffraction halos rather than sharp Bragg peaks, indicating a predominantly semi-crystalline or amorphous structure. The XRD patterns of the two sides of the Janus membrane closely follow those of the corresponding single-component electrospun membranes, suggesting that tetracycline incorporation and Janus assembly do not introduce new crystalline phases or significantly alter the polymer chain packing.

**Fig. S5.** TGA curves of PA8F electrospun membrane, PA10F electrospun membrane, Janus membrane without/with TC.

All samples exhibit similar thermal decomposition behaviour, characterised by a minor initial mass loss below ~150 °C, followed by a dominant single-step degradation at higher temperatures. The main decomposition occurs between approximately 420 and 500 °C, indicating good thermal stability of both PA8F- and PA10F-based electrospun membranes.

The TGA curves of the Janus membranes with and without TC are nearly overlapping throughout the entire temperature range, with no additional low-temperature degradation step observed for the TC-containing sample. This behaviour suggests that the incorporation of TC does not measurably alter the thermal stability of the Janus membrane, which can be attributed to the relatively low TC loading compared to the polymer matrix. At 600 °C, the Janus membranes show a slightly higher residual mass than the single-component electrospun membranes, while the difference between the TC-loaded and TC-free Janus membranes remains negligible.


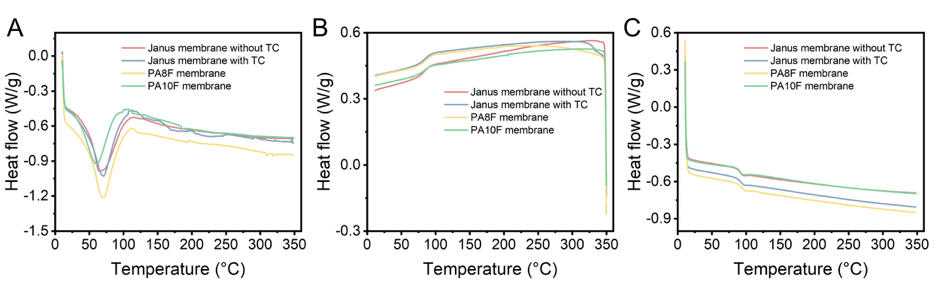


**Fig. S6.** DSC curves of PA8F electrospun membrane, PA10F electrospun membrane, Janus membrane without/with tetracycline (TC). (A) first heating from 10 to 350 °C, (B) cooling from 350 to 10 °C, and (C) second heating from 10 to 350 °C (10 °C min⁻¹).

First heating (Fig. S6A). All electrospun samples show a pronounced low-temperature thermal event between approximately 50–110 °C, appearing as a broad endothermic feature superimposed on the baseline. This feature is most intense for the PA8F electrospun membrane, consistent with its stronger water affinity and higher uptake of physically bound moisture in the nanofibrous structure. By comparison, the PA10F electrospun membrane shows a noticeably weaker response in the same temperature window, in line with its more hydrophobic character. Both Janus membranes display intermediate behaviour.

Above ~120 °C, no sharp melting endotherm is evident up to 350 °C for any sample; instead, the curves gradually approach a similar baseline. During cooling from 350 to 10 °C, the curves are broadly similar and do not show a strong, well-defined crystallisation peak (Fig. S6B). Only subtle changes in slope/weak features are observed, suggesting limited or slow crystallisation kinetics in these electrospun membranes. The two Janus curves remain highly overlapped throughout cooling.

After removing thermal history, the second heating scans show highly reproducible baselines with a weak thermal transition/inflection around ~90 °C, consistent with the glass-transition region of these semi-aromatic polyamides (Fig. S6C). Importantly, the Janus membrane with TC and without TC are nearly indistinguishable across the entire second heating range. The absence of an additional transition associated with TC is attributed to the low TC loading relative to the polymer mass, which is insufficient to measurably perturb the bulk thermal response in DSC.

**Molecular dynamics simulation details and additional analyses**

**Coarse-grain mapping and force field**

The CG model was based on the corresponding state correlations of the SAFT-γ Mie formalism [9], with each repeat unit of PA8F and PA10F mapped into distinct interaction sites corresponding to their chemical motifs. The mapping, as seen in Fig. S7, consisted of:

- Four acetamide beads (**AMC**),
- Three furan beads (**FUR**),
- Three (n-octane in PA8F) or four (n-decane in PA10F) alkyl spacer beads (**A08** and **A10**, respectively).


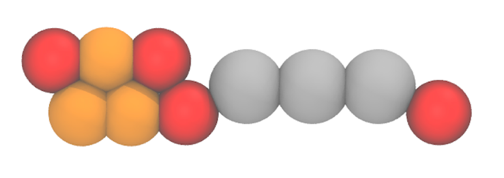


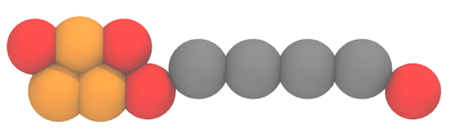


**Fig. S7** (Top) PA8F and (bottom) PA10F CG representations as described in the text.

The CG non-bonded parameters, number of beads (m), repulsive exponent (λr), diameter (σ), energy (ε) for these bead types were calculated using the SAFT-γ Mie correlations [9] (see Table S2) from representative surrogate molecules (acetamide, furan, n-octane, n-decane) and water [10], without further fitting. This choice enables predictive modelling that maintains the thermodynamic consistency of the underlying equation-of-state framework. All molecular dynamics simulations were conducted in Gromacs (v2018.8) which allows user tabulated potentials [11,12].

**Table S2.** SAFT-γ Mie parameters

| **CG Bead** | **m** | **lr** | **e [K]** | **s [nm]** |
| --- | --- | --- | --- | --- |
| **A08** | 3 | 16.22 | 334.29 | 0.422 |
| **A10** | 4 | 15.30 | 317.41 | 0.407 |
| **AMC** | 2 | 12.79 | 447.21 | 0.353 |
| **FUR** | 3 | 10.52 | 210.19 | 0.312 |
| **W** | 1 | 8.40 | 378.87 | 0.292 |

**System preparation**

Polymer slabs were constructed by randomly packing 1,000 repeat units of either PA8F or PA10F in a periodic elongated box and equilibrating under isothermal-isobaric (NPT) conditions to obtain the bulk density. After equilibration, the systems were simulated in the NVT ensemble to generate slabs >7 nm thick (in the *z*-axis), enabling a well-defined interior bulk region and two independent surfaces. Fig. S8 depicts the equilibrated cell for PA8F with dimensions *L_x_=L_y_=* 9 nm and *L_z_=*18 nm. Production runs of 2 ns were performed, during which density profiles and surface compositions were analysed.


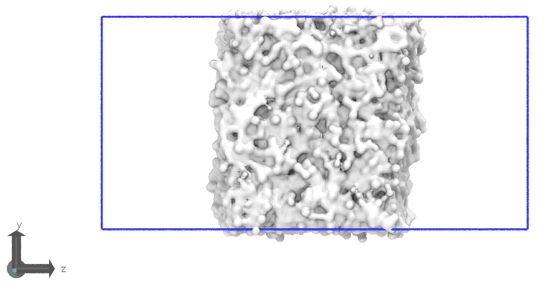


**Fig. S8** PA8F simulation cell oriented in the z-axis from left to right, vacuum, surface, bulk, surface, vacuum.

**Water infiltration simulations**

A nanoscopic droplet of 1,728 coarse-grained water beads was positioned near the polymer surface, and the time evolution of water infiltration was monitored. Production runs of 2 ns were performed, during which water percolation was analysed.

Coarse-grained molecular simulations based on a SAFT-γ Mie model were used to compare the surface hydration behaviour of PA8F and PA10F membranes. The simulations reveal that small molecular differences in polymer architecture led to pronounced differences in surface chemical composition and hydration pathways. PA8F exposes more polar functional groups at its interface and enables more rapid water infiltration, whereas PA10F presents a more hydrophobic surface enriched in aliphatic carbon.

These results support the experimentally observed differences in wettability and highlight the molecular origins of chain-length dependent hydrophilicity in these polyamides. The approach demonstrates how coarse-grained modelling can provide predictive insights into polymer surface properties and guide the rational design of functional membrane materials.

**UV–vis calibration curve for tetracycline quantification**

**
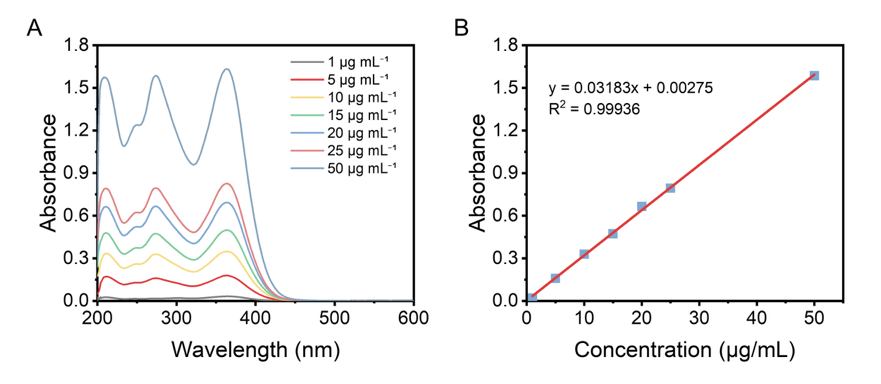
**

**Fig. S9**. (A) UV–vis absorption spectra and (B) calibration curve of tetracycline (TC) obtained from standard TC solutions with concentrations of 1, 5, 10, 15, 20, 25, and 50 μg mL⁻¹.

The UV–vis calibration curve of tetracycline (TC) was constructed using standard TC solutions with concentrations of 1, 5, 10, 15, 20, 25, and 50 μg mL⁻¹. The absorbance at 275 nm, corresponding to the characteristic absorption peak of TC, increased linearly with concentration. Linear regression analysis yielded a correlation coefficient of R² = 0.99936, demonstrating excellent linearity over the investigated concentration range. This calibration curve was used for quantitative determination of TC concentration in the release studies.


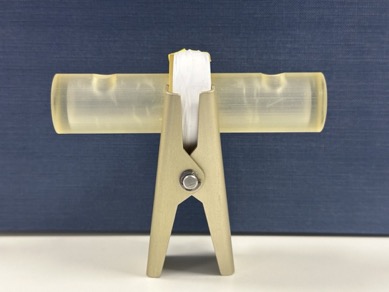


**Fig. S10.** Photograph of the custom-built directional drug release setup used to evaluate asymmetric tetracycline transport across the Janus membrane.

The device consists of two opposing cylindrical chambers with an inner diameter of 1.5 cm, defining the effective membrane area exposed to the release medium. The Janus membrane was fixed securely between the chambers using waterproof PTFE tape to prevent leakage and ensure physical separation of the two compartments during release experiments.


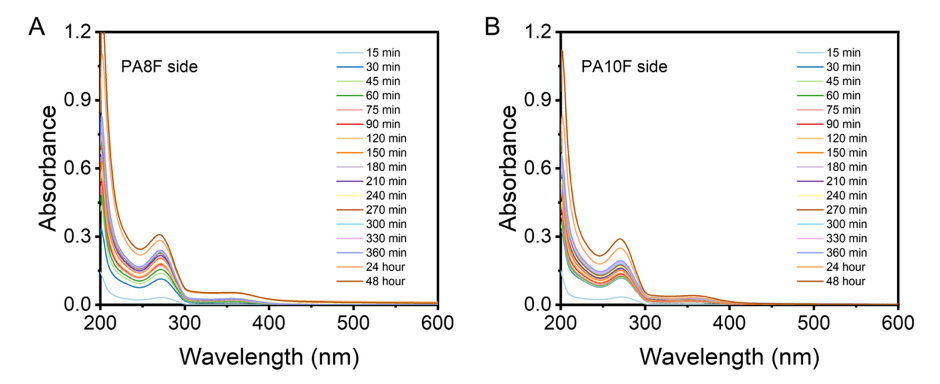


**Fig. S11.** Time-dependent UV-vis absorption spectra of PBS solutions collected from each side of the Janus membrane using a custom 3D-printed dual-chamber device, (A) PA8F side and (B) PA10F side.

**
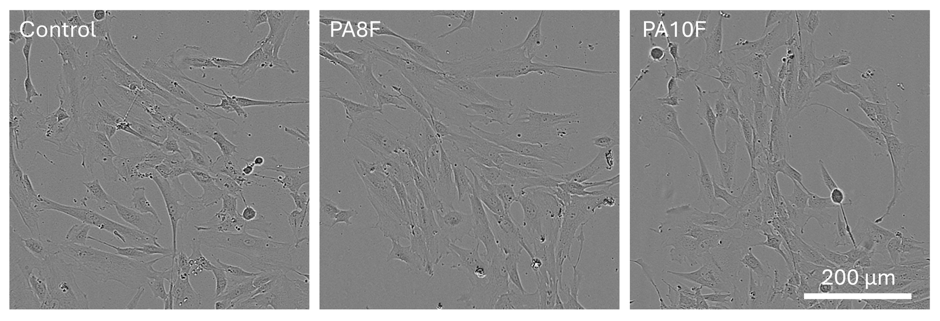
**

**Fig. S12.** Representative brightfield images of fibroblasts after 72 h exposure to control medium or membrane-conditioned media from PA8F and PA10F.

**Minimum Inhibitory Concentration (MIC) testing of Tetracycline**

The minimum inhibitory concentration (MIC) of tetracycline against *Pseudomonas aeruginosa* (PAO1) and *Staphylococcus aureus* (H560) was determined using a broth microdilution method. A stock solution of tetracycline (128 μg mL⁻¹) was prepared in sterile MilliQ water and serially two-fold diluted to a final concentration of 0.03125 μg mL⁻¹ in Luria–Bertani (LB) broth for PAO1 and tryptic soy broth (TSB) for H560.

Overnight bacterial cultures (18 h) were diluted 1:1000 in the corresponding fresh medium. Aliquots of 100 μL bacterial suspension were added to each well of a 96-well microtiter plate containing 100 μL of tetracycline at the designated concentration. Each condition was tested in triplicate. Plates were incubated at 37 °C with shaking, and bacterial growth was monitored by measuring the optical density at 600 nm (OD₆₀₀) over 18 h using a BMG Labtech microplate reader.

Growth inhibition curves were generated by plotting OD₆₀₀ values at 18 h against the logarithm of tetracycline concentration using Origin software. MIC₅₀ and MIC₉₀ values were determined by sigmoidal fitting of the dose–response curves, as shown in Fig. 6A and Fig. 6B.


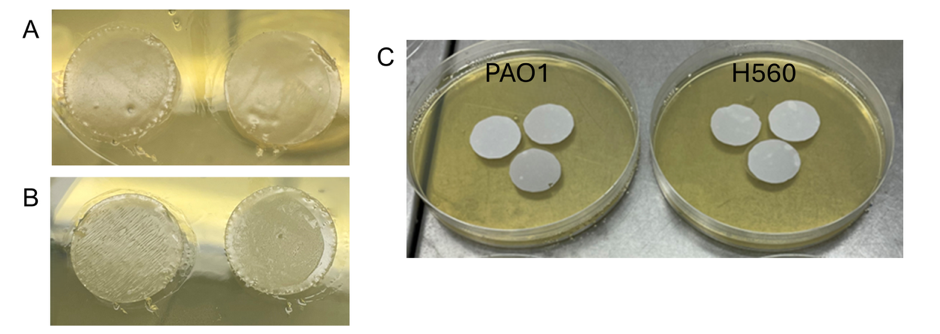


**Fig. S13.** 8-hour colony biofilms of (A) PAO1 and (B) H560 on nano-porous polycarbonate membranes; (C) Janus membranes on colony biofilms at 8-hour time point.

**Quantifying tetracycline release from Janus membrane into water**

Janus membrane with transparent tape backing was cut into 3 square pieces of 2.5 × 2.5 cm^2^ in size. Pipette 1 mL of sterilised MilliQ water in each small petri dish and place each Janus membrane on top of water. The petri dish was sealed with parafilm and incubate at 37 °C for 24 hours. After 24 hours of incubation, 200 μL of water from each petri dish was transferred into 96 well plate and perform the MIC assay.

**Fig. S14.** Absorbance vs. half dilution factor of water containing tetracycline released from Janus membrane. Concentration of tetracycline calculated using known MIC_90_ on H560 strain (from Fig. 6B).

Released tetracycline of unknown concentration in water was used to grow the H560 strains and the growth curve of optical density vs half dilution factors was constructed. Using the half dilution factor in which 90% of growth inhibition was achieved, the concentration of tetracycline was calculated in reverse dilution factor using the known MIC_90_ of H560 strain (i.e., 1 μg mL^-1^). 2.56 μg mL^-1^ of tetracycline was released from 1 cm^2^ of Janus membrane.

**Table S3.** Comparison of antibacterial/antibiofilm outcomes reported for selected Janus antibacterial membranes/dressings.

| **Janus system** | **Antibacterial design** | **Antibacterial evaluation model** | **Representative antibacterial performance** | **Ref.** |
| --- | --- | --- | --- | --- |
| PSP/PAN // OXA+P_2_W_18_/PLA Janus electrospun dressing | Dual-agent antibacterial (oxacillin + polyoxometalate synergy) | In vivo wound infection (MRSA-infected mouse wound; CFU from wound) | CFU (counts): Day 10 MRSA < 1.0 × 10⁵ CFU (Janus dressing and OXA/P2W18/PLA) vs > 6.5 × 10⁵ CFU (single-drug fibers or drug-free groups). | [13] |
| CGPT-PCZ Janus nanofibrous membrane | Dual-agent: antibiotic release (TCH) + inorganic contact/ROS (Cu–ZnO) | Agar diffusion (ZOI) | ZOI diameter (mm): Bilayer 30.56 ± 1.46 (S. aureus), 25.32 ± 1.83 (E. coli), 17.69 ± 1.14 (P. aeruginosa). | [14] |
| PLGAV-CuS/PVAM amphiphilic Janus nanofibrous membrane | Antibiotic + photothermal-assisted release (CuS, NIR) | Planktonic antibacterial assay+ in vivo infected wound | % inhibition (bacteriostatic rate): Janus membrane 82.6% (E. coli) and 85.4% (S. aureus); with NIR 96.3% and 97.8%, respectively. | [15] |
| PP // PP/dopamine@30 nm ZnO Janus membrane | Antibacterial surface (ZnO; biofilm inhibition) | Surface-attached biofilm (S. mutans biofilm on membrane, 3 days) | Biofilm coverage area reduced from 73.46% ± 1.58% (PP) to 49.34% ± 2.15% (PP/dopamine@ZnO). | [16] |
| Agar/AgNPs film // electrospun agar/PCL + gallic acid (AP-GA) Janus dressing | Sustained AgNP antibacterial + antioxidant (GA) | Agar diffusion + time-kill (log CFU) + in vivo healing | % inhibition (time-kill): 0.2 mM bilayer reduced bacterial growth by 92% (P. aeruginosa) and 81% (P. vulgaris). | [17] |
| QAS-PU/FC Janus nanofibrous dressing | Contact-killing cationic layer + pro-regenerative collagen inner | In vivo infected wound (qualitative infection suppression + wound healing kinetics) | Wound healing rate (%): Day 3 68 ± 3.3% (QAS-PU/FC) vs 31 ± 14% (gauze). % inhibition: “almost 100% antimicrobial efficiency” (as stated). | [18] |
| PA8F-TC/PA10F bio-based polyamide (This work) | Burst antibiotic delivery (TC in PA8F) + intrinsic wettability asymmetry (materials-by-design) | Colony biofilm + ex vivo tissue infection | CFU (log reduction): ~1 log (PAO1) and ~2 log (H560) in colony biofilm; ~0.5 log reduction in ex vivo porcine burn model vs Janus without TC | This work |

**References**

[1] H. Samadian, S. Zamiri, A. Ehterami, S. Farzamfar, A. Vaez, H. Khastar, M. Alam, A. Ai, H. Derakhshankhah, Z. Allahyari, A. Goodarzi, M. Salehi, Electrospun cellulose acetate/gelatin nanofibrous wound dressing containing berberine for diabetic foot ulcer healing: in vitro and in vivo studies, Sci. Rep. 10 (2020) 8312. https://doi.org/10.1038/s41598-020-65268-7.

[2] S.S. Sharaf, A.M. El-Shafei, R. Refaie, A.A. Gibriel, R. Abdel-Sattar, Antibacterial and wound healing properties of cellulose acetate electrospun nanofibers loaded with bioactive glass nanoparticles; in-vivo study, Cellulose. 29 (2022) 4565–4577. https://doi.org/10.1007/s10570-022-04570-1.

[3] F. Xu, H. Wang, J. Zhang, L. Jiang, W. Zhang, Y. Hu, A facile design of EGF conjugated PLA/gelatin electrospun nanofibers for nursing care of in vivo wound healing applications, J. Ind. Text. 51 (2020) 420S-440S. https://doi.org/10.1177/1528083720976348.

[4] S. Khosravimelal, M. Chizari, B. Farhadihosseinabadi, M. Moosazadeh Moghaddam, M. Gholipourmalekabadi, Fabrication and characterization of an antibacterial chitosan/silk fibroin electrospun nanofiber loaded with a cationic peptide for wound-dressing application, J. Mater. Sci. Mater. Med. 32 (2021) 114. https://doi.org/10.1007/s10856-021-06542-6.

[5] P.-H. Chiu, Z.-Y. Wu, C.-C. Hsu, Y.-C. Chang, C.-M. Huang, C.-T. Hu, C.-M. Lin, S.C. Chang, H.-J. Hsieh, C.-A. Dai, Enhancement of antibacterial activity in electrospun fibrous membranes based on quaternized chitosan with caffeic acid and berberine chloride for wound dressing applications, RSC Adv. 14 (2024) 34756–34768. https://doi.org/10.1039/D4RA05114A.

[6] N. Charernsriwilaiwat, T. Rojanarata, T. Ngawhirunpat, P. Opanasopit, Electrospun chitosan/polyvinyl alcohol nanofibre mats for wound healing, Int. Wound J. 11 (2014) 215–222. https://doi.org/10.1111/j.1742-481X.2012.01077.x.

[7] S. Ullah, M. Hashmi, M.Q. Khan, D. Kharaghani, Y. Saito, T. Yamamoto, I.S. Kim, Silver sulfadiazine loaded zein nanofiber mats as a novel wound dressing, RSC Adv. 9 (2019) 268–277. https://doi.org/10.1039/C8RA09082C.

[8] Q. Wang, S. Zhang, J. Jiang, S. Chen, S. Ramakrishna, W. Zhao, F. Yang, S. Wu, Electrospun radially oriented berberine-PHBV nanofiber dressing patches for accelerating diabetic wound healing, Regen. Biomater. 11 (2024) rbae063. https://doi.org/10.1093/rb/rbae063.

[9] A. Mejía, C. Herdes, E.A. Müller, Force Fields for Coarse-Grained Molecular Simulations from a Corresponding States Correlation, Ind. Eng. Chem. Res. 53 (2014) 4131–4141. https://doi.org/10.1021/ie404247e.

[10] C. Herdes, T.S. Totton, E.A. Müller, Coarse grained force field for the molecular simulation of natural gases and condensates, Fluid Phase Equilib. 406 (2015) 91–100. https://doi.org/10.1016/j.fluid.2015.07.014.

[11] M.J. Abraham, T. Murtola, R. Schulz, S. Páll, J.C. Smith, B. Hess, E. Lindahl, GROMACS: High performance molecular simulations through multi-level parallelism from laptops to supercomputers, SoftwareX. 1–2 (2015) 19–25. https://doi.org/10.1016/j.softx.2015.06.001.

[12] B. Abraham, M. J.; van der Spoel, D.; Lindahl, E.; Hess, GROMACS User Manual version 2018.8, 2019. www.gromacs.org.

[13] X. Zhang, R. Lv, L. Chen, R. Sun, Y. Zhang, R. Sheng, T. Du, Y. Li, Y. Qi, A Multifunctional Janus Electrospun Nanofiber Dressing with Biofluid Draining, Monitoring, and Antibacterial Properties for Wound Healing, ACS Appl. Mater. Interfaces. 14 (2022) 12984–13000. https://doi.org/10.1021/acsami.1c22629.

[14] C. Liu, W. Wu, X. Li, J. Li, Y. Zhang, B. Li, T. Jiao, Engineering of electrospun Janus nanofibrous membranes with synergistic antibacterial strategy via dual-agent incorporation, Colloids Surfaces A Physicochem. Eng. Asp. 728 (2026) 138700. https://doi.org/10.1016/j.colsurfa.2025.138700.

[15] Y. Shi, M. Zhou, S. Zhao, H. Li, W. Wang, J. Cheng, L. Jin, Y. Wang, Janus amphiphilic nanofiber membranes synergistically drive antibacterial and anti-inflammatory strategies for skin wound healing, Mater. Des. 227 (2023) 111778. https://doi.org/10.1016/j.matdes.2023.111778.

[16] Y. Guo, Q. Wang, G. Sun, Y. Zheng, Bioinspired Janus Membrane with Dopamine-ZnO Coating for Antibacterial Filtration in Oral Applications, Polymers (Basel). 17 (2025) 1356. https://doi.org/10.3390/polym17101356.

[17] K. Rathore, D. Upadhyay, N. Verma, A.K. Gupta, S. Matheshwaran, S. Sharma, V. Verma, Asymmetric Janus Nanofibrous Agar-Based Wound Dressing Infused with Enhanced Antioxidant and Antibacterial Properties, ACS Appl. Bio Mater. 7 (2024) 7608–7623. https://doi.org/10.1021/acsabm.4c01184.

[18] J. Song, Z. Gao, Y. Zhang, Y. Li, Y. Zhao, H. Yan, X. Jiang, H. Zheng, Y. Zhang, A. Yao, C. Li, G. Hou, X. Xie, M. Zhang, Bioinspired electrospun janus nanofibrous dressing for synergistic antibacterial activity and tissue regeneration, Front. Microbiol. 17 (2026). https://doi.org/10.3389/fmicb.2026.1788110.
